# Supplementary material for: Key transcriptional effectors of the pancreatic acinar phenotype and oncogenic transformation
Source: PLoS One. 2023 Oct 5;18(10):e0291512. doi: 10.1371/journal.pone.0291512 (PMC10553828; doi:10.1371/journal.pone.0291512)
Supplement: S1 Fig — (PDF) [file pone.0291512.s001.pdf]

Analysis Summary

Identification of dTFs

Identify all sequence-specific DNA-binding transcription factors expressed in the normal pancreas by RNA-seq

↓

Select TF genes with expression >1 rpkm (>1 mRNA per acinar cell). See Fig S2 for list of top 70.

↓

Select TF genes with most restricted expression (<10 mouse tissues/organs of 34 total; NCBI EST analyses)

↓

Select TF genes required for developmental decisions and preliminary evidence for maintance of acinar differentiation

↓

4 most prominent dTF candidates

Ptf1a *Nr5a2* *Foxa2* *Gata4*

Conditional gene inactivations (cKOs) selectively in adult pancreatic acinar cells

↓

Define '**direct control**' of gene transcription by the dTFs

'Affected' genes: RNAseq identification of mRNAs that increase or decrease after dTF gene inactivation

'Bound' genes: genes associated with an acinar cell enhancer (an ARD) containing a bound dTF
ARDs are regions of chromatin with activated H3K4me2 marks and bound RNA polymerase II

'Directly Regulated' genes: genes bound by the dTF at a gene-associated ARD and affected by the dTF cKO.

↓

Functional importance of each candidate dTF determined by the behavior of dTF 'Regulated' genes

Evaluate effects of cKOs on the major differentiation functions of acinar cells by RNAseq

1. Loss of acinar cell identity
   Qualitative and quantitative changes in differentiation status
   Gained expression of genes normally restricted to nonpancreatic cell-types
2. Decreased expression of the genes for the 35 hallmark acinar secretory enzymes
3. Protein production
   Effects on components of the translation apparatus and regulators of protein synthesis optimization
   Quantitative effects on the total rate of protein synthesis
   Effects on genes for modification, processing, transport and exocytosis of the secretory proteins
4. Status of the powerful anabolic metabolism that fuels massive acinar protein synthesis
5. Maintenance of the low level of cell division and DNA replication that maintains acinar cell mass
6. Effects of the loss of acinar differentiation on the resistance to Kras-driven oncogenesis

S1 Fig. Decision tree to identify the four differentiation TFs and the flow/rationale for analyses of the roles they play in key cellular processes that define pancreatic acinar differentiation status and identity.
