# Supplementary material for: Key transcriptional effectors of the pancreatic acinar phenotype and oncogenic transformation
Source: PLoS One. 2023 Oct 5;18(10):e0291512. doi: 10.1371/journal.pone.0291512 (PMC10553828; doi:10.1371/journal.pone.0291512)
Supplement: S2 Fig — (PDF) [file pone.0291512.s002.pdf]

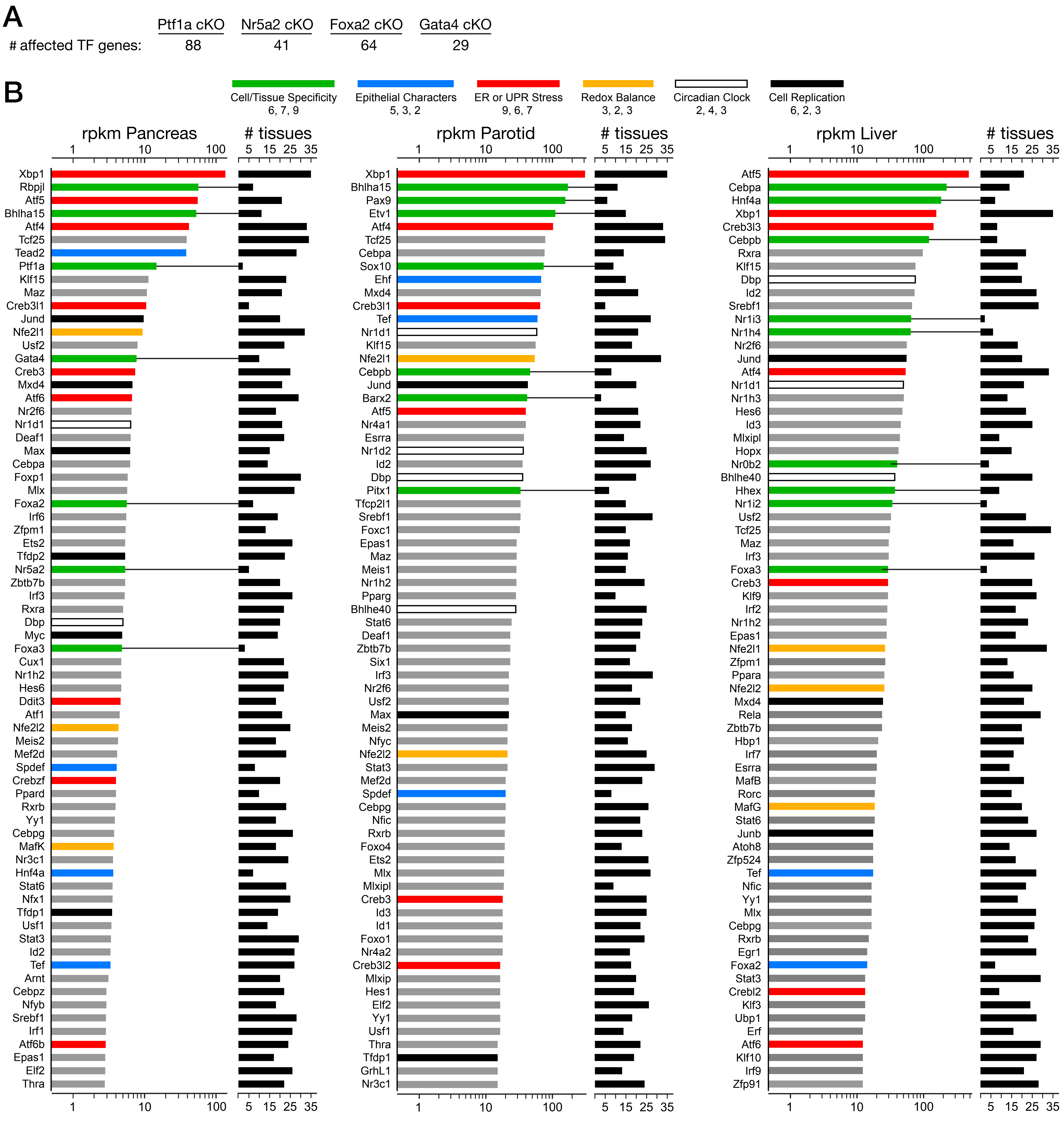

S2 Figure. The seventy highest expressed transcription factors for the mouse pancreas, parotid and liver. **A**. Numbers of genes for pancreatic sequence-specific DNA-binding transcription factors (TFs) affected by each of the four cKOs. **B**. TF genes expressed in the pancreas, parotid and liver ranked according to the the rpkms for their mRNAs derived from RNA-seq analyses. Color coding: TFs associated with specific cellular characteristics: *green*, cell or tissue specific gene transcription; *blue*, epithelial characters; *red*, ER or UPR stress; *orange*, maintenance of reduction/oxidation balance; *white*, the circadian clock; *black*, cellular replication. The associated numbers indicate the number of TFs in each class for pancreas, parotid and liver, respectively. The thin horizontal black lines link cell type-specific TFs to the number of adult mouse tissues/organs (out of a total of 35) in which their expression was detected by expressed-sequence-tag analyses (NCBI, Unigene EST's).

S2 Figure. The seventy highest expressed transcription factors for the mouse pancreas, parotid and liver. **A**. Numbers of genes for pancreatic sequence-specific DNA-binding transcription factors (TFs) affected by each of the four cKOs. **B**. TF genes expressed in the pancreas, parotid and liver ranked according to the the rpkms for their mRNAs derived from RNA-seq analyses. Color coding: TFs associated with specific cellular characteristics: *green*, cell or tissue specific gene transcription; *blue*, epithelial characters; *red*, ER or UPR stress; *orange*, maintenance of reduction/oxidation balance; *white*, the circadian clock; *black*, cellular replication. The associated numbers indicate the number of TFs in each class for pancreas, parotid and liver, respectively. The thin horizontal black lines link cell type-specific TFs to the number of adult mouse tissues/organs (out of a total of 35) in which their expression was detected by expressed-sequence-tag analyses (NCBI, Unigene EST's).
