# Supplementary material for: Key transcriptional effectors of the pancreatic acinar phenotype and oncogenic transformation
Source: PLoS One. 2023 Oct 5;18(10):e0291512. doi: 10.1371/journal.pone.0291512 (PMC10553828; doi:10.1371/journal.pone.0291512)
Supplement: S3 Fig — (PDF) [file pone.0291512.s003.pdf]

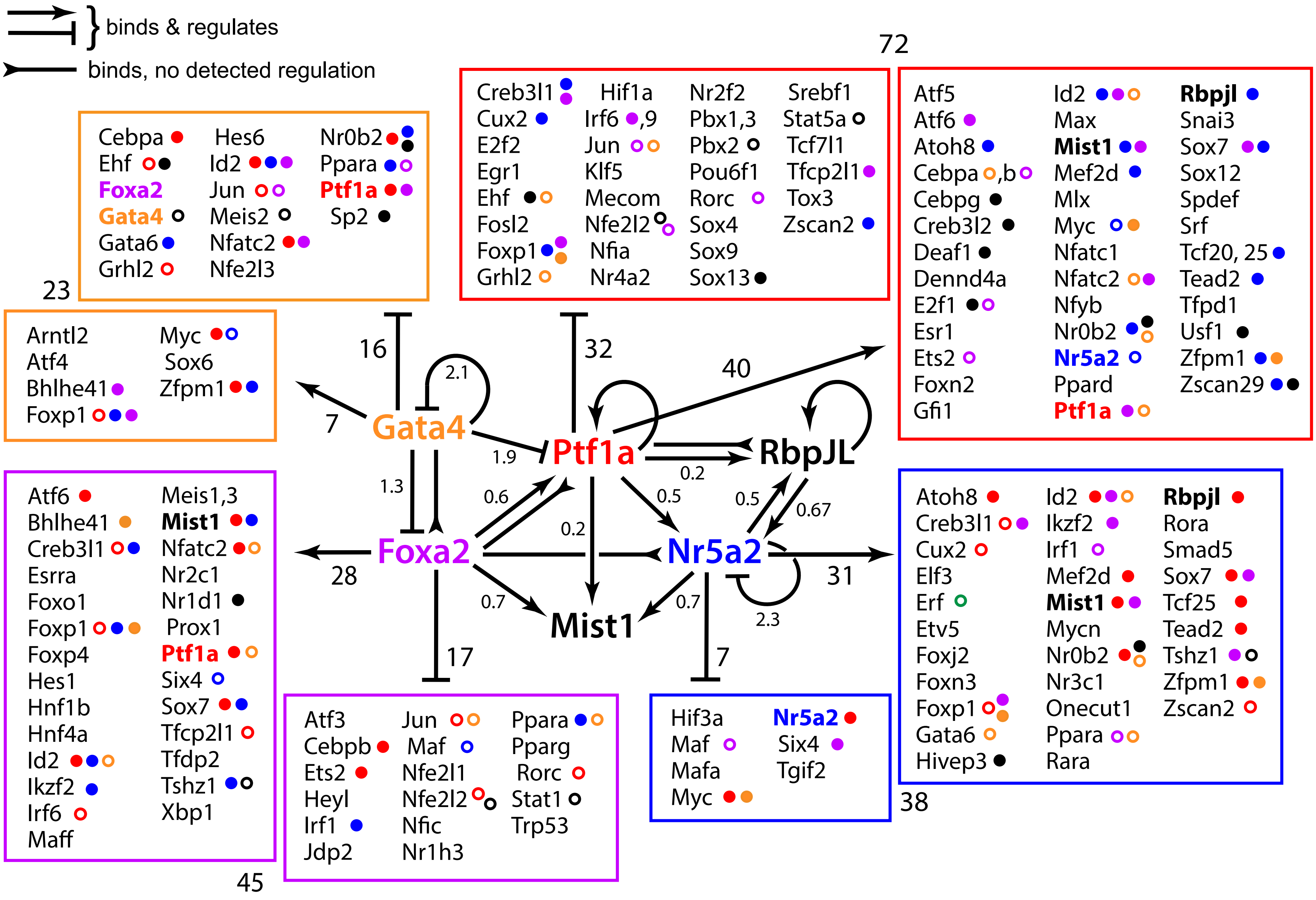

S3 Figure. Regulatory interactions among the dTFs and their control of genes encoding other DNA-binding, sequence-specific transcription factors. Center: numbers in smaller font indicate the fold-changes caused by the cKO of individual dTFs; the numbers in larger font indicate the number of target genes affected. The colored circles within the targeted gene boxes indicate activation by a dTF (solid) or suppression (open), judged by the effects of the individual dTF-cKOs on TF mRNA levels. Only instances of direct regulation based on dTF binding in an ARD are shown here. Also included are the two scaling TFs, Mist1/bHLHa15 and Rbpjl.
