# Supplementary material for: Key transcriptional effectors of the pancreatic acinar phenotype and oncogenic transformation
Source: PLoS One. 2023 Oct 5;18(10):e0291512. doi: 10.1371/journal.pone.0291512 (PMC10553828; doi:10.1371/journal.pone.0291512)
Supplement: S4 Fig — (PDF) [file pone.0291512.s004.pdf]

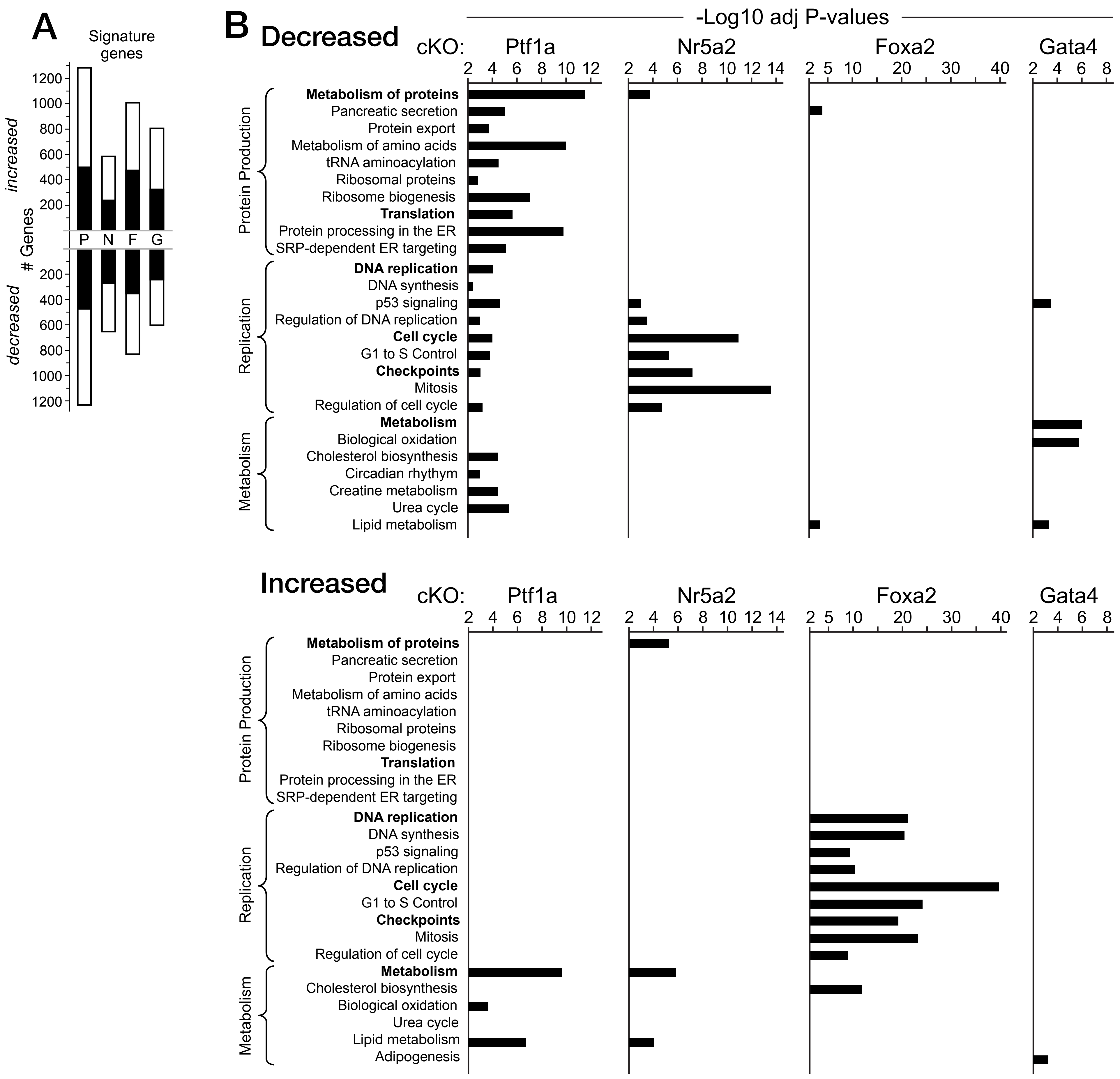

S4 Figure. The sets of signature genes for each cKO confirm the selective regulatory responsibilities of the individual dTFs. Signature gene profiles (Robinson et al., *Bioinformatics* **26**:139, 2010) confirm Ptf1a activation of genes for protein production as well as Foxa2 suppression of replication genes, and uncover stimulatory effects of Nr5a2 on genes of replication. **A.** Total numbers of signature genes selected for >1.5-fold (open bars) and >2-fold (filled bars) changes in mRNA levels. **B**. Cellular pathways from CPDB analyses of the sets of genes indicated in panel A for >1.5-fold change. Note that the Foxa2 scale differs.
