# Supplementary material for: Key transcriptional effectors of the pancreatic acinar phenotype and oncogenic transformation
Source: PLoS One. 2023 Oct 5;18(10):e0291512. doi: 10.1371/journal.pone.0291512 (PMC10553828; doi:10.1371/journal.pone.0291512)
Supplement: S5 Fig — (PDF) [file pone.0291512.s005.pdf]

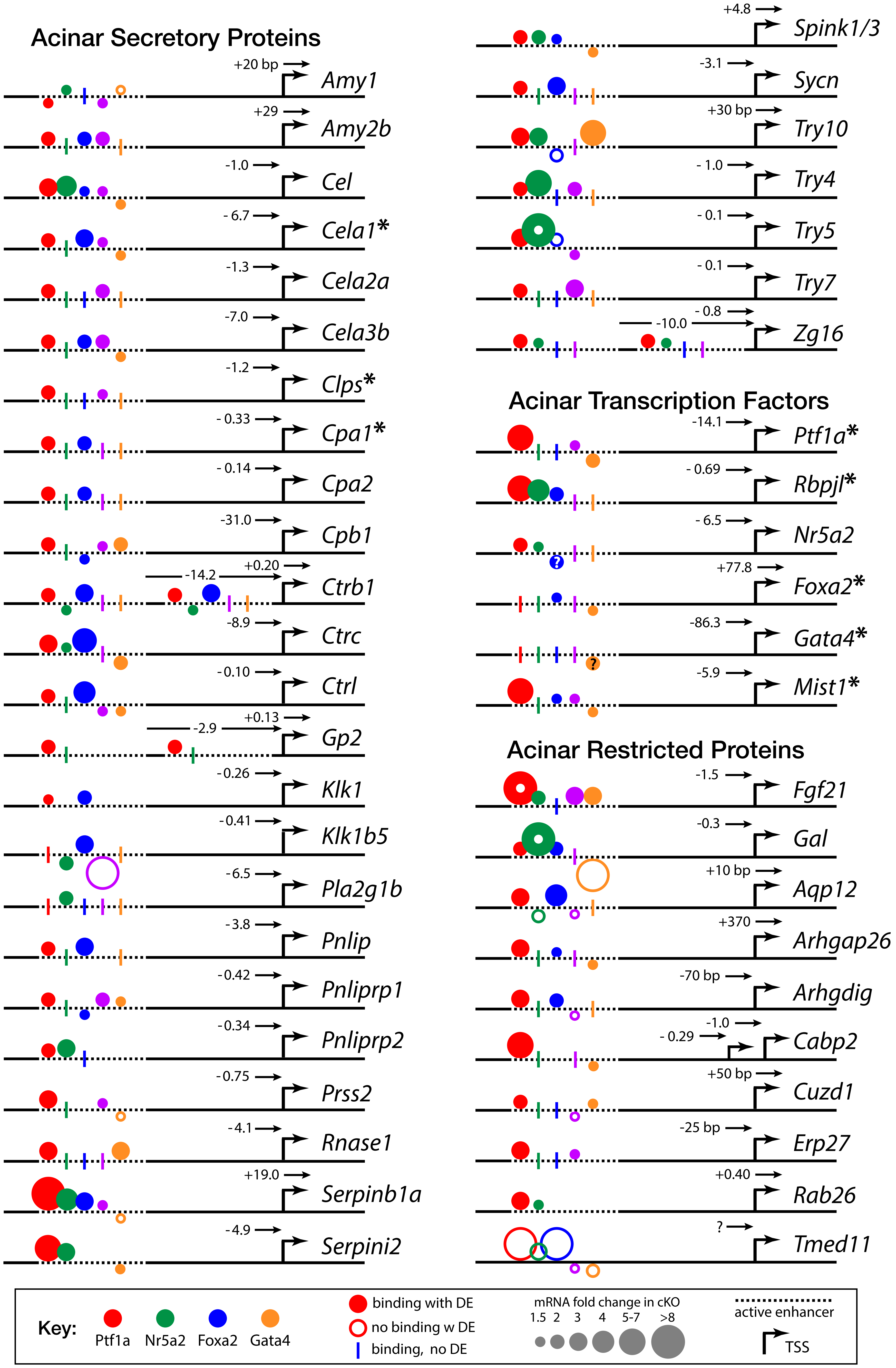

S5 Figure. Correlation of the binding of dTFs (colored circles) to active enhancers (dashed areas) with the effects of dTF-cKOs on the expression of individual acinar restricted genes. *Dashed lines* indicate ARDs based on the presence of RNAPII and histone H3K4dimethylation. Filled circles indicate binding of color-coded dTFs to enhancers. The diameter of the circle represents the fold-change in mRNA level for the corresponding dTF-cKO. Circles above the gene line indicate activation by a dTF (i.e., mRNA levels decreased in the cKO); circles below the line are possible repression (mRNA levels increased). Open circles indicate the fold change for instances without dTF binding. Vertical bars confirm the binding of a dTF when there was no effect on the mRNA level for the corresponding cKO. The distances between enhancers and TSS are indicated. *asterisks*, acinar specificity of the enhancer previously verified by transgenesis. *Tmed11* is an example of cKO effects without evidence of an active enhancer or binding of dTFs. The *Klk1b11*, *Prss1*, and *Prss3* genes have very low mRNA levels, and though affected by dTF cKOs, have no detectable dTF binding and neither marker of an Active Regulatory Domain.
