# Supplementary material for: Key transcriptional effectors of the pancreatic acinar phenotype and oncogenic transformation
Source: PLoS One. 2023 Oct 5;18(10):e0291512. doi: 10.1371/journal.pone.0291512 (PMC10553828; doi:10.1371/journal.pone.0291512)
Supplement: S6 Fig — (PDF) [file pone.0291512.s006.pdf]

| dTF ChIP | Enriched auxiliary motif(s) | Auxiliary factor-enhanced enrichment of cellular processes |
|---|---|---|
| **Ptf1a** | Nfi half-site | Pathways: Aspects of Intermediary metabolism, Cancer<br>GO: no additional |
| **Nr5a2** | Nfi dimer and Nfic | Pathways: Cancer, Apoptosis, aspects of Intermediary metabolism<br>GO: Regulation of DNA-binding TF activity; Actomyosin structural organization |
| **Foxa2** | Nfi:Foxa and Nfic | Pathways: Cell junctions<br>GO: PDGF receptor signaling |
| **Gata4** | Nfix | Pathways: Apoptosis<br>GO: PDGF receptor signaling; ER stress-induced apoptotic signaling |
| | Thap11 (dimer) | Pathways: Transcription. GO: no additional |
| | Znf143 (dimer) | Pathways: Transcription. GO: no additional |
| | Atf1 | Pathways: no additional. GO: no additional |
| | Elk1 | Pathways: Translation. GO: Translation |

**S6 Figure.** Pathway enrichment for genes with ARDs bound by dTFs and potential cofactors. Auxiliary TF binding site motifs in dTF peaks were identified by de novo discovery using HOMER. Only pathways enriched for genes selectively associated with ARDs containing both dTF binding and an auxiliary TF binding motif are listed. *Pathways*, from gene analysis by WebGestalt (Wang J et al., 2017. *Nucleic Acids Res* **45**:W130-W137). *GO*, cellular functions from gene ontology using GREAT analysis (McLean CY et al., 2010. *Nat Biotechnol* **28**:495-501) of peaks.
