# Supplementary material for: Key transcriptional effectors of the pancreatic acinar phenotype and oncogenic transformation
Source: PLoS One. 2023 Oct 5;18(10):e0291512. doi: 10.1371/journal.pone.0291512 (PMC10553828; doi:10.1371/journal.pone.0291512)
Supplement: S7 Fig — (PDF) [file pone.0291512.s007.pdf]

A

dTF cKO	Total secretory protein mRNA rpkm (% control)	% Control Protein Synthesis Rate*
C- Control	732K (100)	(100)
P- Ptf1a	488K (66.6)	49.7
N- Nr5a2	811K (111)	51.6
F- Foxa2	444K (60.6)	86.7
G- Gata4	644K (88.0)	63.6

* from text Figure 3F

B

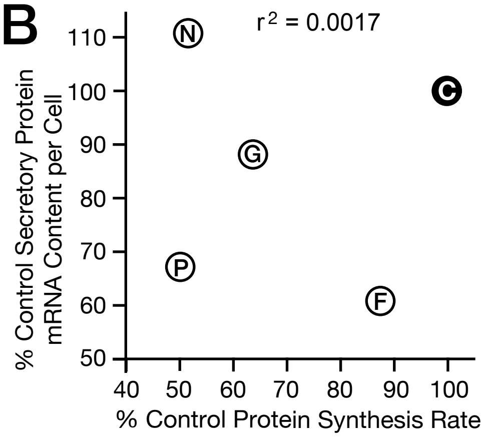

C

cKO	# of 130 Myc Hallmark genes	# of 28 Myc Hallmark genes of protein synthesis
Ptf1a	44/11/55 (42)	16/0/16 (57)
Nr5a2	14/8/12 (9.2)	3/0/3 (11)
Foxa2	4/19/23 (18)	0/1/1 (3.5)
Gata4	14/3/17 (13)	3/1/4 (14)

Down/**Up**/**Total** (%)

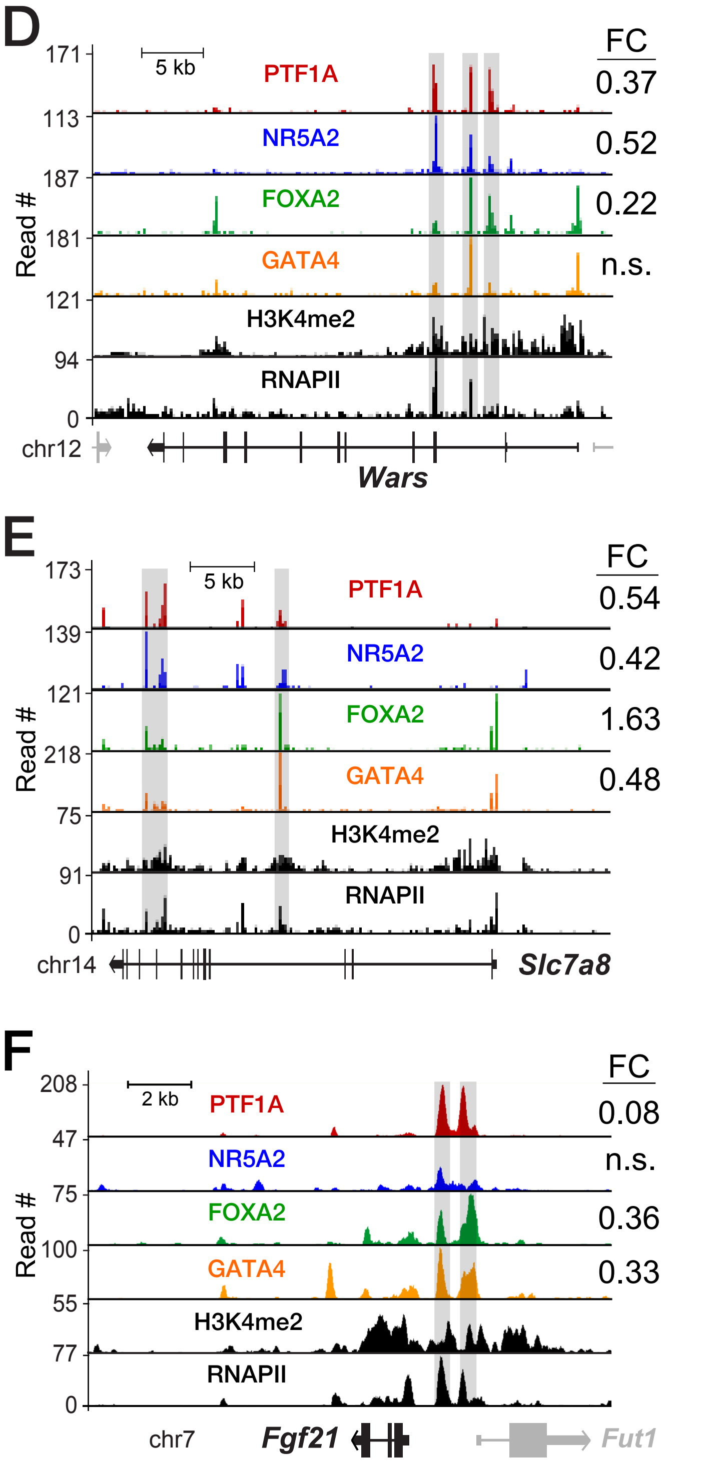

S7 Figure. Regulation of protein synthesis. **A.** Comparison of the decreased size of the secretory protein mRNA population (which composes >90% of the total acinar mRNA population) and the measured rates of protein synthesis in vitro. The total number of mRNA molecules per diploid PAC is estimated between 0.5 and 1 million (B.P. Davis et al., *J Biol Chem* **267**:26070, 1992; PMID 1464618). **B**. No correlation between total amounts of secretory protein mRNA and measured rates of protein synthesis in dTF-deficient pancreases. cKOs: P, Ptf1a; N, Nr5a2; F, Foxa2; G, Gata4; C, control. **C**. Effects of the cKOs on the set of 130 Myc Hallmark genes, which have been shown to be bound and up-regulated by Myc (K.I. Zeller et. al., *Genome Biol* **4**:R69, 2003; GSEA M6506). **D**-**F**. ChIPseq profiles for the four dTFs binding to *Wars* (**D**), *Slc7a8* (**E**), and Fgf21 (**F**) loci; FC, fold-change of the corresponding mRNA for each dTF-cKO; *n.s.*, no significant change (fdr >0.01). Shaded bars, ARDs.
