# Supplementary material for: Key transcriptional effectors of the pancreatic acinar phenotype and oncogenic transformation
Source: PLoS One. 2023 Oct 5;18(10):e0291512. doi: 10.1371/journal.pone.0291512 (PMC10553828; doi:10.1371/journal.pone.0291512)
Supplement: S8 Fig — (PDF) [file pone.0291512.s008.pdf]

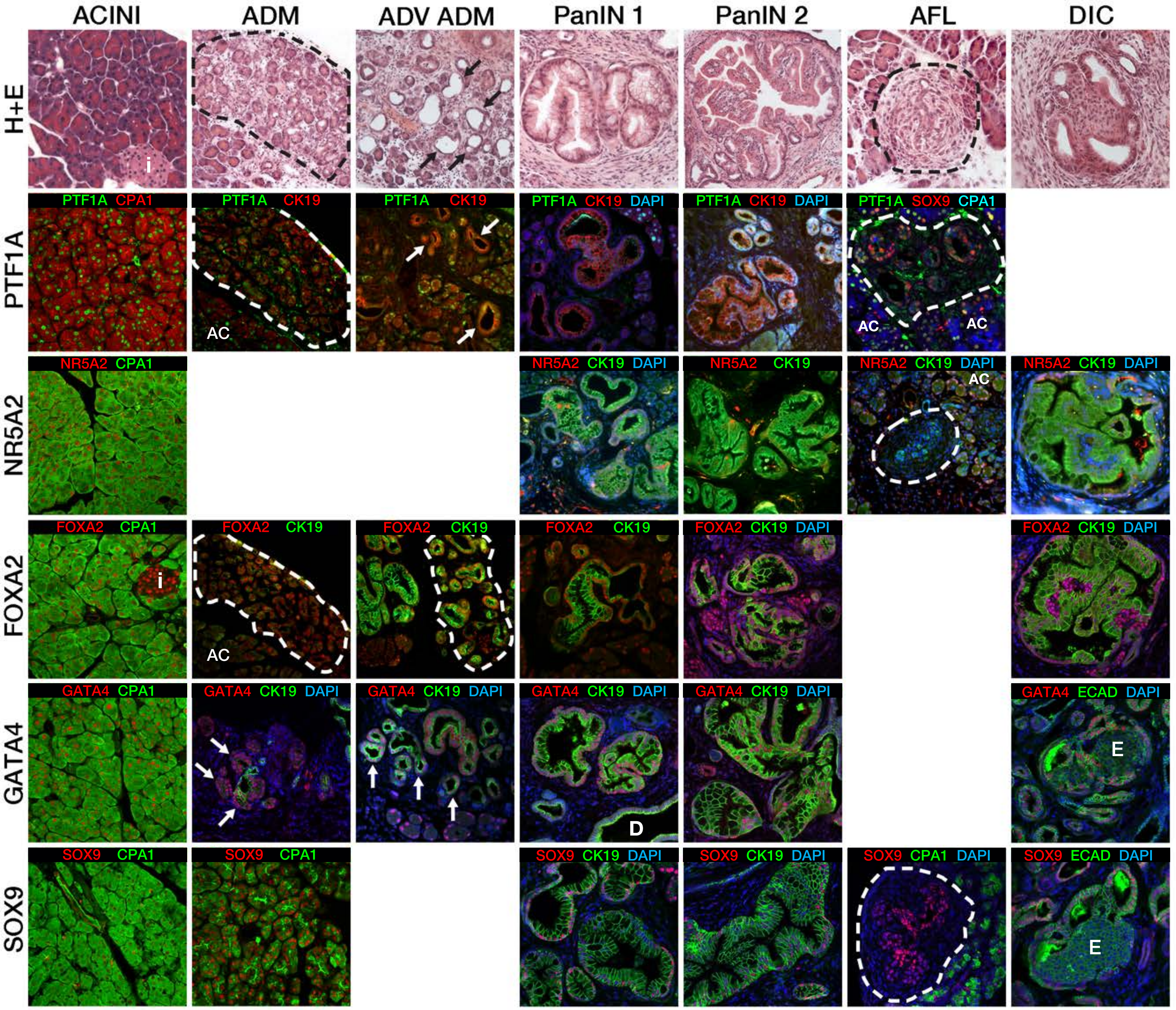

S8 Figure, Part 1. Additional distinguishing immunofluorescent markers of preneoplastic lesions. All images are pancreas with oncogenic Kras expression, except for the acinar images, which are normal (*Ptf1a +/CreERT*). *Arrows* indicate examples of histochemical-stained or antigen-positive cells or groups of cells for the markers listed in the left margins. *Dashed outlines* delimit lesions. Key: *i*, islet; *AC*, acinar cell; *D*, duct; *E*, endocrine compartment of DICs. All confocal and light microscopic images were photographed through a 20X objective. Rows are labeled according to the stain or marker. Comments are limited to the epithelial cells for each type of lesion, except for CD45.**Row H+E** (hematoxylin and eosin): normal acinar cells have a large cytoplasm with extensive basal RER (purple) surrounding a nucleus and a pink apical domain containing zymogen granules; lumina are small. ADM tubules with diminishing acinar morphology, often with still extensive cytoplasm and generally larger lumina. Advanced ADM, extended lumina with thinned single-cell layered epithelia. PanIN-1, huge apical light pink cytoplasm with basal nuclei in single-cell layer convoluted epithelia. **Row Ptf1a**: key acinar dTF is high in >90% normal acinar cells only, present in most ADM cells, absent or low in Adv ADM, and absent in PanINs, AFL and DIC. **Row Nr5a2**: acinar dTF is high in normal acinar cells, low in the majority of PanIN-1 cells, low in a minority of PanIN-2 cells, and absent from AFL, and low in minority of PanIN-like DIC cells. **Row Foxa2**: acinar dTF is high in most acinar and islet cells, ADM, Adv ADM, PanIN-1, PanIN-2, and endocrine and PanIN-like cells of DIC. **Row Gata4**: acinar dTF is present in most acinar, ADM and Adv ADM cells and retained in most PanINs, and PanIN-like DIC cells. **Row Sox9**: marker of pancreatic ADM is present high in duct and centroacinar cells, low in most acinar cells, high in ADM, PanIN, AFL, and PanIN-like DIC cells.

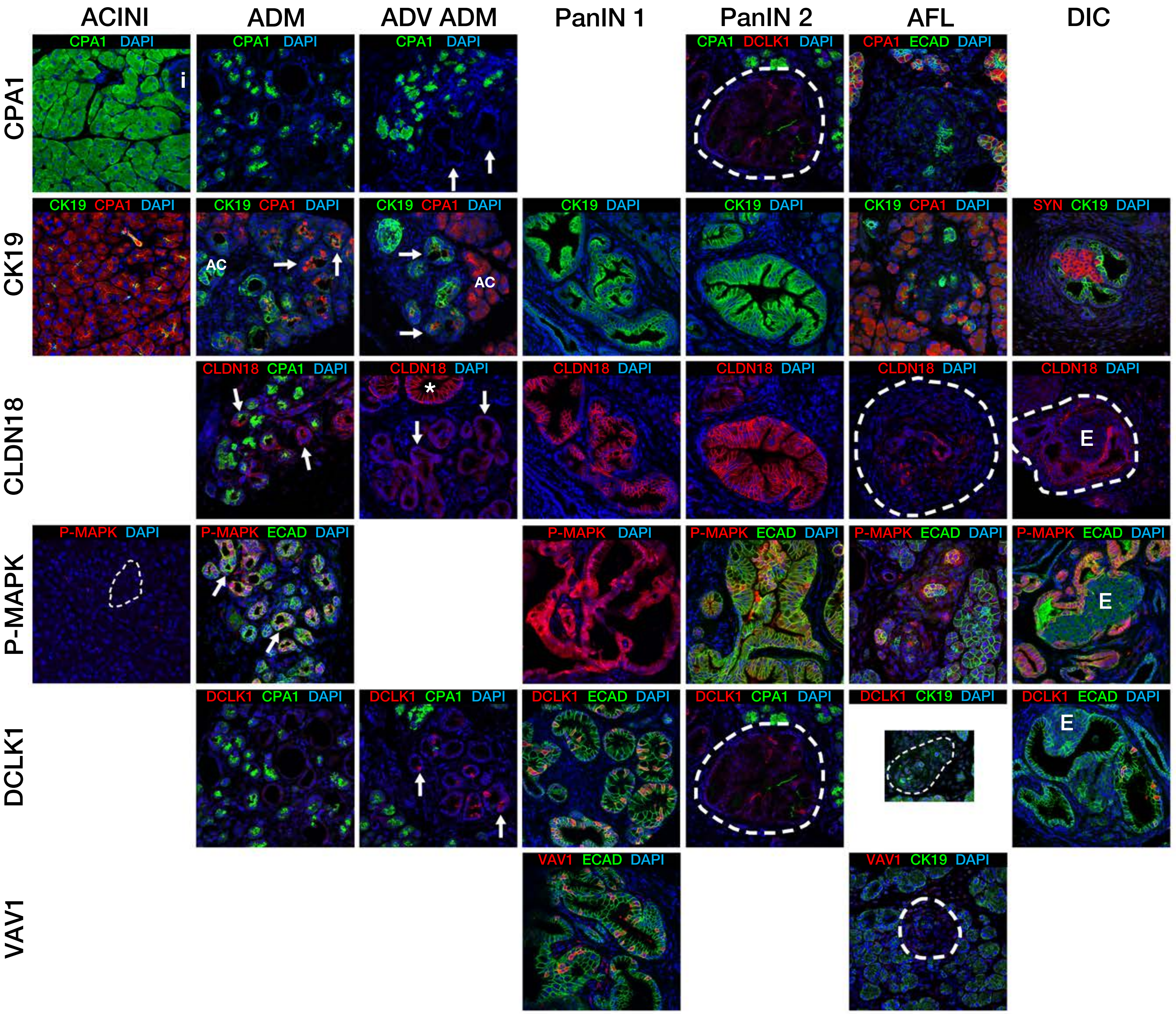

S8 Figure, Part 2.
Row CPA1: acinar differentiation marker is present in acinar cells, lower in ADM cells, but not in cells of Adv ADM, PanINs, AFL or DIC epithelium. **Row CK19**: ductal and ADM marker is present in normal centroacinar, intercalated and intralobular ductal cells but not acinar cells; present in ADM, Adv ADM, PanIN, AFL, and DIC epithelial cells. **Row Cldn18**: preneoplastic marker appears at low level in ADM (* PanIN) and Adv ADM cells, high in PanIN and PanIN-like DIC cells, and partial in AFL cells. **Row P-MAPK**: not present in acinar cells; present in ADM, PanIN-1, frequent with variable levels in PanIN-2, high in most cells of AFL and the PanIN-like DIC cells. **Row DCLK1**: marker of pancreatic Tuft cells is absent in acinar cells (not shown), ADM and AFL; appears in isolated cells with Tuft cell morphology in Adv ADM, PanINs, and PanIN-like DIC cells. **Row VAV1**: a second marker verified presence of Tuft cells in PanIN lesions, but not in AFL.

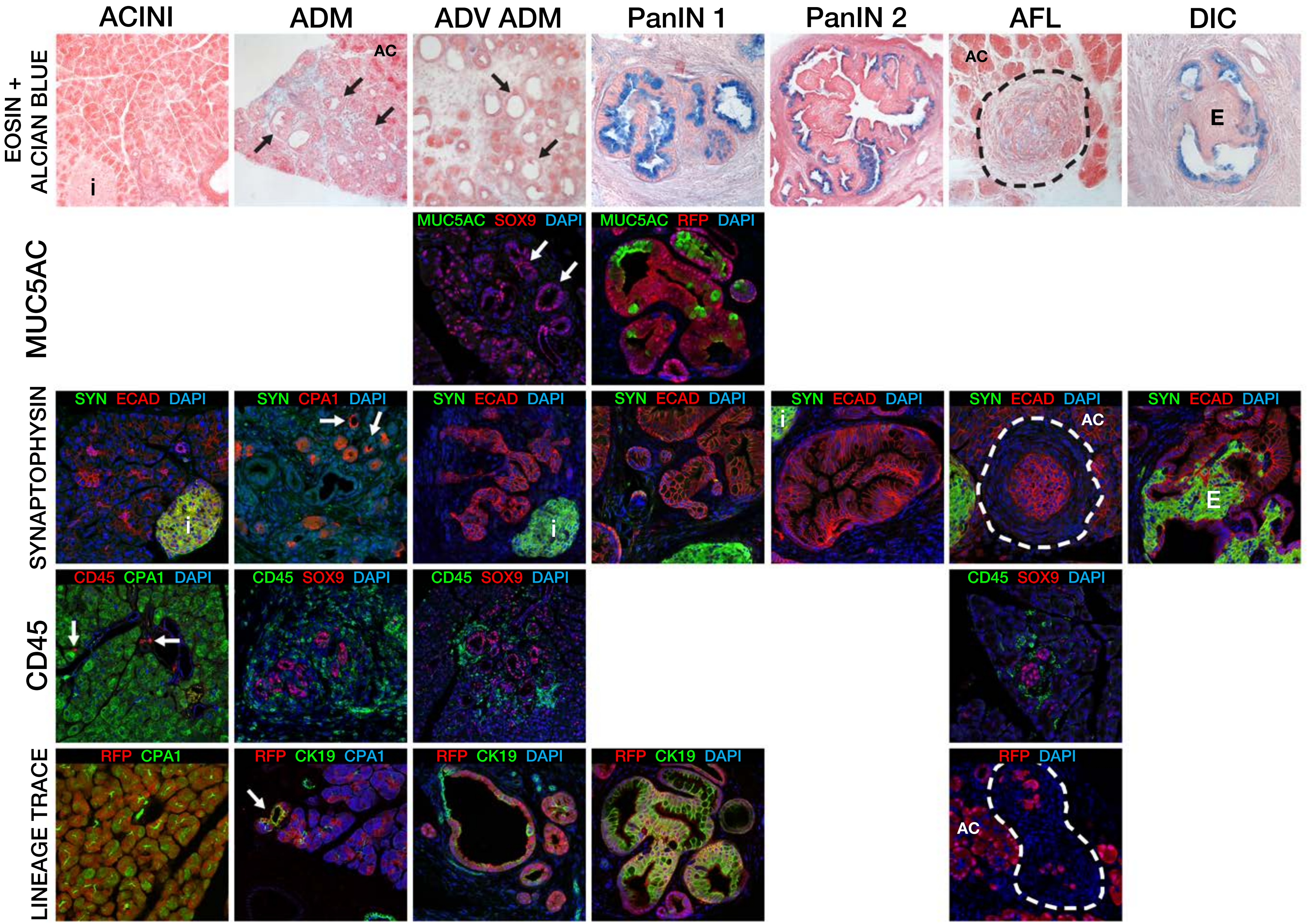

S8 Figure, Part 3.
Row Eosin + Alcian Blue: staining for acidic mucin first appears in the thin apical cytoplasm of Adv ADM tubules, enhances in the expanded apical cytoplasm of PanINs, absent in AFL, and present in PanIN-like DIC cells. Note low level staining of stroma for each type of lesion. **Row MUC5AC**: acidic mucin marker of PanIN lesions first appears in PanIN1 epithelium. **Row SYN**: marker of neuro-endocrine cells is present in normal islets (*i*) and the endocrine compartment of DIC; coincides with insulin staining (text Figure 8N). Note solid ECAD+ epithelial core of AFL. **Row CD45**: CD45+ immune cells are present occasionally in normal pancreatic tissue, large numbers with SOX9+ ADM and Adv ADM, and AFL. **Row LINEAGE TRACE**: Rosa26 tdTomato-marked acinar cells lineage-trace to ADM, Adv ADM, PanINs and AFL.
