# Supplementary material for: Key transcriptional effectors of the pancreatic acinar phenotype and oncogenic transformation
Source: PLoS One. 2023 Oct 5;18(10):e0291512. doi: 10.1371/journal.pone.0291512 (PMC10553828; doi:10.1371/journal.pone.0291512)
Supplement: S1 Table — (PDF) [file pone.0291512.s009.pdf]

S1 Table. Efficiency of floxed-dTF deletion by Ptf1a^{CreERT}

cKO/Antibody		Genotype	Mouse ID	TF+/CPA1+	% CPA1+ being mTF-	Avg %
**Ptf1a**	Ptf1a-cKO	Ptf1aCreERT/fl	3Ptf1aFC-44-2	80/975	91.8	91.8*
	Control	Ptf1aCreERT/+	3Ptf1aFC-44-3	362/374	3.2	7.7%
		"	"	667/759	12.1	
**Nr5a2**	Nr5a2-cKO	Nr5a2fl/fl; Ptf1aCreERT/+	LPFCb 12-2	39/2199	98.2	95.7
		"	LPFCb 12-6	125/1226	89.8	
		"	LPFCb 13-7	24/3056	99.2	
	Control	Ptf1aCreERT/+	Ptf1aFC-44-5	2309/2353	1.9	2.6
		"	Ptf1aFC-44-7	2980/3037	1.9	
		"	Ptf1a C-10-1	565/588	3.9	
**Foxa2**	Foxa2-cKO	FoxA2fl/fl; Ptf1aCreERT/+	FPFC-24-2	0/886	100	99.8
		"	FPFC-24-12	8/1735	99.5	
		"	FPFC-25-1	3/1846	99.8	
	Control	Ptf1aCreERT/+	Ptf1aFC-44-5	2405/2435	1.2	3.0
		"	Ptf1aFC-44-7	2062/2157	4.4	
		"	Ptf1a C-10-1	462/478	3.3	
**Gata4**	Gata4-cKO	Gata4fl/fl; Ptf1aCreERT/+	G3	128/2329	94.5	94.3
		"	G9	129/2142	94	
		"	GPFC-26-2	84/1518	94.5	
	Control	Ptf1aCreERT/+	Ptf1aFC-44-5	2965/3031	2.2	1.9
		"	Ptf1aFC-44-7	2750/2781	1.1	
		"	Ptf1a C-10-1	546/560	2.5	

*Our previous study showed that the analysis of three Ptf1a-cKO pancreases had an average gene deletion of 88% (Hoang et al. 2016 *Mol Cell Biol* **36**:3033 PMID:27697859).
