# Supplementary material for: Key transcriptional effectors of the pancreatic acinar phenotype and oncogenic transformation
Source: PLoS One. 2023 Oct 5;18(10):e0291512. doi: 10.1371/journal.pone.0291512 (PMC10553828; doi:10.1371/journal.pone.0291512)
Supplement: S2 Table — (DOC) [file pone.0291512.s010.doc]

**S2 Table.** Gene sets for pathways in text Figures 1, 3, 4 and 5.

**Figure 1G Gene Sets**

**34 Pancreatic Acinar Secretory Protein Genes** (Hoang et al. *Mol Cell Biol* **36**:3033, 2016)**.**

Amy1, Amy2a4,3,4,5, Amy2b, Cel, Cela1, Cela2a, Cela3b, Clps, Cpa1, Cpa2, Cpb1, Ctrb1, Ctrc, Ctrl, Gp2, Klk1, Klk1b11, Klk1b5, Pla2g1b, Pnlip, Pnliprp1, Pnliprp2, Prss1, Prss2, Prss3, Rnase1, Serpinb1a, Serpini2, Spink3, Sycn, Try10, Try4, Try5, Try7/2210010C04Rik, Zg16.

**23 Pancreatic Acinar Restricted Genes** (this study). Aqp8, Aqp12, Asb16, C2cd4b, Cabp1, Cabp2, Cuzd1, Dagla, Erp27, Ffar2, Fgf21, Gal, Hsd17b13, Noc2L, Otud3, Pdia2, Rab26, Rph3al, Slc39a5, Tff2, Tinag, Tmed11, Tmed6.

**159 Pancreatic Transcription Factor Genes** (this study)**.** Assembled for all of the DNA-binding TF genes with altered expression for at least one cKO. ArntL2, Atf3, Atf4, Atf5, Atf6, Atf6b, Atoh8, Bhlha15/Mist1, Bhlhe41, Blzf1, Cebpa, Cebpb, Cebpg, Cebpz, Creb3l1, Creb3l2, Crebzf, Cux2, Ddit3/Chop, Deaf1, Dennd4a, E2f1, E2f2, Egr1, Ehf, Elf3, Erf, Esr1, Esrra, Ets2, Etv5, Fosl2, Foxa2, Foxj2, Foxk2, Foxn2, Foxn3, Foxo1, Foxo3, Foxp1, Foxp4, Gata4, Gata6, Gfi1, Gli1, GrhL1, GrhL2, Hes1, Hes6, Hes7, Hif1a, HeyL, Hif3a, Hnf1a, Hnf1b, Hnf4a, Id1, Id2, Ikzf2, Irf3, Irf1, Irf6, Irf7, Irf9, Jdp2, Jun, Jund, Klf5, Maf, Mafa, Maff, Mafb, Max, Mecom, Mef2d, Meis1, Meis2, Meis3, Mga, Mlx, , Myc, Mycn, Nfat5, Nfatc1, Nfatc2, Nfe2l1, Nfe2l2, Nfe2l3, Nfia, Nfic, Nfkb2, Nfxl1, Nfyb, Nkrf, Nr0b2, Nr1d1, Nr1h3, Nr1h4, Nr2c1, Nr2f6, Nr3c1, Nr4a1, Nr4a2, Nr5a2/Lrh1, Onecut1, Pbx1, Pbx2, Pbx3, Pou6f1, Ppara, Ppard, Pparg, Prox1, Ptf1a, Rara, Rarg, Rbpjl, Relb, Rest, Rfx2, Rora, Rorc, Six4, Smad5, Snai3, Sox12, Sox13, Sox18, Sox4, Sox6, Sox7, Sox9, Sp2, Spdef, Srebf1, Srf, Stat1, Stat5a, Tcf20, Tcf25, Tcf7, Tcf7L1, Tead2, Tead3, Tfcp2l1, Tfdp1, Tfdp2, Tgif2, Tox, Tox3, Trp53, Tshz1, Usf1, Usf2, Xbp1, Zfpm1/Fog1, Zfp64, Zscan2, Zscan29.

**300 Top Acinar Differentiation Genes** (Hoang et al. *Mol Cell Biol* **36**:3033, 2016)**.** The 300 genes with greatest increased expression during late pancreatic differentiation (i.e., 18.5 dpc to 8 weeks after birth). 1190003J15Rik, 1700028J19Rik, 1810018F18Rik, 1810022K09Rik, 2210010C04Rik, 2410127L17Rik, 2610005L07Rik, 2900097C17Rik, 3230401D17Rik, 4930415O20Rik, 9930111J21Rik1, A130077B15Rik, Aass, Actg1, Agmo, Ahcy, Ap1s3, Ap2m1, Aph1a, Apoo, Arg2, Arl5b, Armcx3, Asb11, Atp12a, Atp5g1, Atp5g2, Atp5h, Atp5l, AW112010, Azgp1, B3galt5, Bloc1s2a, Bmpr1b, Btf3, Btf3l4, C1rl, C4b, Capza1, Car1, Car3, Car4, Cbx3, Ccl11, Cdk2ap1, Ceacam1, Cela1, Ces1e, Cfd, Cilp, Cir1, Ckmt1, Clca3, Clic5, Cmah, Cml1, Cox7c, Cpa1, Cpb1, Ctrc, Ctrl, Cuzd1, Cxcl14, Cycs, Cyp2e1, Cyp39a1, Cyp3a13, Cytip, Dbp, Ddo, Ddx3y, Dmbt1, Dnajb6, Dnajc19, Dpep1, Dusp26, Eci3, Eef1a1, Eef1g, Eif2s3x, Eif2s3y, Eif4a1, Erh, Ero1lb, Errfi1, Fam103a1, Fam134b, Fam189a2, Fam60a, Fau, Fcgbp, Fetub, Fgf21, Fgl2, Gabarapl2, Galnt6, Gapdh, Gda, Ggh, Glul, Gm10845, Gm129, Gm17821, Gna14, Gp2, Gps1, Gpx4, Gstm2, Gucd1, H2afz, Hadhb, Hamp2, Hax1, Hgfac, Hmgb1, Hmgcs2, Hnmt, Hnrnpa3, Hnrnpf, Hsd11b1, Hspa8, Ifi205, Igj, Immp1l, Impdh2, Itih2, Itih4, Iyd, Klf9, Klk1, Klk1b11, Klk1b24, Klk1b3, Klk1b4, Klk1b5, Kpna2, Kprp, Lars2, Llph, Lpin1, Lypd8, Lyz1, Mgat4c, Mpc1, Mptx1, Mrpl23, Mrps33, Mt2, Muc13, Muc2, Muc5ac, Myl6, N4bp2l1, Naca, Ncoa4, Ndufab1, Ndufb4, Nfasc, Nhp2l1, Nr1d2, Nrbf2, Nsa2, Nudt21, Odc1, Olfr613, Olfr856-ps1, Pah, Pam16, Pde6a, Pdk4, Pgam1, Pgk1, Phgdh, Piga, Pigr, Pin4, Pla2g1b, Plin4, Pnlip, Pnliprp2, Polr2k, Ppia, Ppp1cc, Prodh, Prss2, Psenen, Psma3, Ptger3, Ptma, Rasgef1b, Reg1, Reg2, Reg3a, Reg3b, Reg3d, Reg3g, Rfwd2, Rn45s, Rnase1, Rpl10, Rpl10a, Rpl11, Rpl12, Rpl13, Rpl14, Rpl15, Rpl17, Rpl18, Rpl18a, Rpl19, Rpl21, Rpl23, Rpl23a, Rpl24, Rpl27, Rpl27a, Rpl28, Rpl29, Rpl3, Rpl31, Rpl32, Rpl35, Rpl35a, Rpl36, Rpl36a, Rpl36al, Rpl37, Rpl37a, Rpl38, Rpl39, Rpl41, Rpl5, Rpl6, Rpl7, Rpl7a, Rpl9, Rps10, Rps12, Rps13, Rps16, Rps17, Rps18, Rps19, Rps2, Rps23, Rps24, Rps25, Rps27a, Rps28, Rps29, Rps3a1, Rps4x, Rps6, Rps7, Rps8, Rpsa, Runx2, Sarnp, Scd1, Sec61g, Sesn1, Sgk1, Slc19a2, Slc25a35, Slc26a3, Slc30a2, Slc7a8, Sord, Srsf12, Steap4, Styx, Sumo2, Tcea1, Tfb2m, Tff2, Tgm3, Thrsp, Timm23, Timm8a1, Tmc5, Tmed2, Tmem235, Tom1, Tpt1, Trp53inp1, Try4, Tsnax, Tufm, U2af1, Uba52, Ube2s, Ube2v1, Upp2, Usmg5, Usp2, Uty, Vdac3, Vipr1, Zbtb16, Zfp488, Zg16.

**524 Protein Synthesis and Processing** **Genes** (nonredundant compilation for Translation, Protein Processing in the ER, 3’UTR Translational Regulation, tRNA aminoacylation, Nonsense Mediated Decay, Protein Export, N-linked Glycosylation, Amino Acid Transport, SRP-dependent Import into the ER, Peptide Chain Elongation, Translation Termination, and Translation Initiation pathways from Reactome and KEGG). 1700009N14Rik, 2610020H08Rik, Aars, Aars2, Aimp2, Alg1, Alg10b, Alg11, Alg12, Alg14, Alg2, Alg3, Alg5, Alg6, Alg8, Alg9, Amfr, Atf4, Atf6, Atf6b, Atxn3, AU018091, B4galt1, B4galt2, B4galt3, B4galt4, B4galt5, B4galt6, Bag1, Bag2, Bak1, Bax, Bcap31, Bcl2, Bms1, Calr, Canx, Capn1, Capn2, Cars, Cars2, Casc3, Casp12, Cirh1a, Ckap4, Cryaa, Cryab, Csnk2a1, Csnk2a2, Csnk2b, Cul1, Dad1, Dars, Dars2, Ddit3, Ddost, Derl1, Derl2, Derl3, Dkc1, Dnaja1, Dnaja2, Dnajb1, Dnajb11, Dnajb12, Dnajb2, Dnajc1, Dnajc10, Dnajc3, Dnajc5, Dnajc5b, Dnajc5g, Dolk, Dolpp1, Dpagt1, Dpm1, Dpm2, Dpm3, Drosha, Ears2, Edem1, Edem2, Edem3, Eef1a1, Eef1b2, Eef1d, Eef1e1, Eef1g, Eef2, Eef2k, Eftud1, **Eif1a**, **Eif1ad**, Eif1ax, **Eif2a**, Eif2ak1, Eif2ak2, Eif2ak3, Eif2ak4, Eif2b1, Eif2b2, Eif2b3, Eif2b4, Eif2b5, **Eif2c2**, Eif2s1, Eif2s2, Eif2s3x, Eif2s3y, Eif3a, Eif3b, Eif3c, Eif3d, Eif3e, Eif3f, Eif3g, Eif3h, Eif3i, Eif3j1, Eif3j2, Eif3k, Eif4a1, Eif4a2, Eif4b, Eif4e, Eif4ebp1, Eif4g1, Eif4h, Eif5, **Eif5a**, Eif5b, Eif6, Emg1, Eprs, Erlec1, Ern1, Ero1l, Ero1lb, Erp29, Etf1, Fars2, Farsa, Farsb, Fau, Fbl, Fbll1, Fbxo2, Fbxo6, Fcf1, Fut8, Ganab, Gar1, Gars, Gfpt1, Gfpt2, Gmppa, Gmppb, Gnl2, Gnl3, Gnl3l, Gnpnat1, Gspt2, Gtpbp4, Hars, Hars2, Heatr1, Herpud1, Hsp90aa1, Hsp90ab1, Hsp90b1, Hspa1a, Hspa1b, Hspa1l, Hspa2, Hspa4l, Hspa5, Hspa8, Hspbp1, Hsph1, Hyou1, Iars, Iars2, Immp1l, Immp2l, Imp3, Imp4, Kars, Lars, Lars2, Lman1, Lman1l, Lman2, Lsg1, Magoh, Man1a, Man1a2, Man1b1, Man1c1, Man2a1, Man2a2, Manea, Map2k7, Map3k5, Mapk10, Mapk8, Mapk9, Mar6, Mars, Mars2, Mbtps1, Mbtps2, Mcfd2, Mdn1, Mgat1, Mgat2, Mgat3, Mgat4a, Mgat4b, Mgat4c, Mgat5, Mlec, Mlst8, Mogs, Mphosph10, Mpi, Mtor, Mvd, Nars, Nars2, Nat10, Ncbp1, Ncbp2, Nfe2l2, Ngly1, Nhp2, Nhp2l1, Nmd3, Nob1, Nol6, Nop10, Nop56, Nop58, Nploc4, Nsfl1c, Nvl, Nxf1, Nxf2, Nxf3, Nxf7, Nxt1, Nxt2, Os9, Oxa1l, P4hb, Pabpc1, Park2, Pars2, Pdia3, Pdia4, Pdia6, Pgm3, Plaa, Pmm1, Pmm2, Pop1, Pop4, Pop5, Pop7, Ppa1, Ppa2, Ppp1r15a, Preb, Prkcsh, Pwp2, Qars, Rad23a, Rad23b, Ran, Rars, Rars2, Rasl2-9, Rbm28, Rbm8a, Rbx1, Rcl1, Rexo1, Rexo2, Rft1, Rheb, Riok1, Riok2, Rnf185, Rnf5, Rnps1, Rnr1, Rnr2, Rpl10, Rpl10a, Rpl11, Rpl12, Rpl13, Rpl13a, Rpl14, Rpl17, Rpl18, Rpl18a, Rpl19, Rpl22, Rpl23, Rpl23a, Rpl24, Rpl26, Rpl27, Rpl27a, Rpl29, Rpl3, Rpl30, Rpl32, Rpl34, Rpl35, Rpl35a, Rpl37, Rpl37a, Rpl38, Rpl39, Rpl3l, Rpl4, Rpl5, Rpl6, Rpl7, Rpl8, Rpl9, Rplp0, Rplp1, Rplp2, Rpn1, Rpn2, Rpp25, Rpp25l, Rpp30, Rpp38, Rpp40, Rps10, Rps11, Rps12, Rps13, Rps14, Rps15, Rps15a, Rps16, Rps17, Rps18, Rps19, Rps2, Rps20, Rps21, Rps23, Rps24, Rps25, Rps27, Rps27a, Rps28, Rps29, Rps3, Rps3a1, Rps4x, Rps5, Rps6, Rps6kb1, Rps7, Rps8, Rps9, Rpsa, Rptor, Rrbp1, Rrp7a, Sar1a, Sar1b, Sars, Sars2, Sbds, Sec11a, Sec11c, Sec13, Sec23a, Sec23b, Sec24a, Sec24b, Sec24c, Sec24d, Sec31a, Sec31b, Sec61a1, Sec61a2, Sec61b, Sec61g, Sec62, Sec63, Sel1l, Sil1, Skp1a, Slc16a10, Slc1a4, Slc1a5, Slc36a1, Slc36a2, Slc38a1, Slc38a2, Slc38a4, Slc38a5, Slc3a1, Slc3a2, Slc43a1, Slc43a2, Slc6a12, Slc6a14, Slc6a15, Slc6a18, Slc6a19, Slc6a20a, Slc6a20b, Slc6a6, Slc7a1, Slc7a10, Slc7a11, Slc7a2, Slc7a3, Slc7a5, Slc7a6, Slc7a7, Slc7a8, Slc7a9, Smg1, Smg8, Smg9, Spata5, Spcs1, Spcs2, Spcs3, Srp14, Srp19, Srp54a, Srp54b, Srp54c, Srp68, Srp72, Srp9, Srpr, Srprb, Ssr1, Ssr2, Ssr3, Ssr4, St3gal4, St6gal1, St8sia2, St8sia3, St8sia6, Stt3a, Stt3b, Stub1, Svip, Syvn1, Taf9, Tars, Tars2, Tbl3, Tcof1, Traf2, Tram1, Tusc3, Txndc5, Uap1, Uba52, Ubb, Ubc, Ube2d1, Ube2d2a, Ube2d3, Ube2e1, Ube2e2, Ube2e3, Ube2g1, Ube2g2, Ube2j1, Ube2j2, Ube4b, Ubqln1, Ubqln2, Ubqln3, Ubqln4, Ubqlnl, Ubxn6, Ufd1l, Uggt1, Uggt2, Upf1, Upf2, Upf3a, Upf3b, Utp14a, Utp14b, Utp15, Utp18, Utp6, Vars, Vars2, Vcp, Vimp, Wars, Wars2, Wdr3, Wdr36, Wdr43, Wdr75, Wfs1, Xbp1, Xpo1, Xrn1, Xrn2, Yars, Yars2, Yod1, Zbtb11.

**609 Replication Genes** (nonredundant list combining DNA replication and cell cycle pathways from Reactome and KEGG). 1700123L14Rik, 4921501E09Rik, A730008H23Rik, Aaas, Abcc4, Abl1, Abraxas1, Actr1a, Adap2, Ahctf1, Ajuba, Akap9, Akt1, Akt2, Akt3, Anapc1, Anapc10, Anapc11, Anapc13, Anapc15, Anapc16, Anapc2, Anapc4, Anapc5, Anapc7, Ankle2, Apbb1ip, Arpp19, Atm, Atr, Atrip, Aurka, Aurkb, B9d2, Babam1, Babam2, Bard1, Bin2, Birc5, Blm, Blzf1, Bora, Brca1, Brcc3, Brip1, Btrc, Bub1, Bub1b, Bub3, C1qc, C3, Capg, Ccna1, Ccna2, Ccnb1, Ccnb2, Ccnb3, Ccnd1, Ccnd2, Ccnd3, Ccne1, Ccne2, Ccnh, Ccp110, Cd37, Cd4, Cd84, Cdc14a, Cdc14b, Cdc16, Cdc20, Cdc23, Cdc25a, Cdc25b, Cdc25c, Cdc26, Cdc27, Cdc42, Cdc45, Cdc6, Cdc7, Cdca5, Cdca8, Cdk1, Cdk2, Cdk4, Cdk5rap2, Cdk6, Cdk7, Cdkn1a, Cdkn1b, Cdkn1c, Cdkn2a, Cdkn2b, Cdkn2c, Cdkn2d, Cdt1, Cenpa, Cenpc1, Cenpe, Cenpf, Cenph, Cenpi, Cenpj, Cenpk, Cenpl, Cenpm, Cenpn, Cenpo, Cenpp, Cenpq, Cenps, Cenpt, Cenpu, Cenpw, Cenpx, Cep131, Cep135, Cep152, Cep164, Cep192, Cep250, Cep290, Cep41, Cep57, Cep63, Cep70, Cep72, Cep76, Cep78, Cetn2, Chek1, Chek2, Ckap5, Cks1b, Clasp1, Clasp2, Clip1, Clspn, Cnep1r1, Cntrl, Cop1, Creb3l2, Crebbp, Csnk1d, Csnk1e, Ctdnep1, Cul1, Cxcl16, Cytl1, Dbf4, Dctn1, Dctn2, Dctn3, Diaph2, Dkc1, Dna2, Dpyd, Dsn1, Dync1h1, Dync1i1, Dync1i2, Dync1li1, Dync1li2, Dynll1, Dynll2, Dyrk1a, E2f1, E2f2, E2f3, E2f4, E2f5, Elf4, Ensa, Ep300, Ercc6l, Esco1, Esco2, Espl1, Exo1, Fbxl18, Fbxl7, Fbxo5, Fbxw11in11, Fen1, Fgfr1op, Fkbp15, Fkbpl, Foxm1, Fzr1, Gadd45a, Gadd45b, Gadd45g, Gal3st4, Gapt, Gins1, Gins2, Gins3, Gins4, Gm10093, Gm12260, Gm12657, Gm14920, Gm28043, Gm5601, Gm7020, Gm7535, Gmnn, Golga2, Gorasp1, Gorasp2, Gpx1, Gsk3b, Gtse1, H2afb2, H2afb3, H2afj, H2afv, H2afx, H2afz, H3f3a, H3f3b, H3f3c, Haus1, Haus2, Haus3, Haus4, Haus5, Haus6, Haus7, Haus8, Hcls1, Hdac1, Hdac2, Hdac8, Herc2, Hist1h2aa, Hist1h2ab, Hist1h2ac, Hist1h2ad, Hist1h2ae, Hist1h2ag, Hist1h2ah, Hist1h2ai, Hist1h2an, Hist1h2ao, Hist1h2ap, Hist1h2ba, Hist1h2bb, Hist1h2bc, Hist1h2be, Hist1h2bg, Hist1h2bh, Hist1h2bm, Hist1h2bq, Hist1h3a, Hist1h3b, Hist1h3c, Hist1h3d, Hist1h3e, Hist1h3f, Hist1h3g, Hist1h3h, Hist1h3i, Hist1h4a, Hist1h4b, Hist1h4c, Hist1h4d, Hist1h4f, Hist1h4h, Hist1h4i, Hist1h4j, Hist1h4k, Hist1h4m, Hist1h4n, Hist2h2aa1, Hist2h2aa2, Hist2h2ac, Hist2h2be, Hist2h3b, Hist2h3c1, Hist2h3c2, Hist2h4, Hist4h4, Hjurp, Hlx, Hmmr, Hsp90aa1, Hsp90ab, Hsp90ab1, Hus1, Igsf6, Il10ra, Il13ra1, Il18, Incenp, Itgam, Itgax, Itgb2, Itgb3bp, Jak2, Kat5, Kcne3, Kif18a, Kif20a, Kif23, Kif2a, Kif2b, Kif2c, Kmt5a, Knl1, Kntc1, Lcmt1, Lhfpl2, Lig1, Lin37, Lin52, Lin54, Lin9, Lmna, Lmnb1, LOC108167320, LOC108167694, Loxl3, Lpin1, Lpin2, Lpin3, Lyl1, Lyn, Mad1l1, Mad2l1, Mad2l2, Maf, Mapk1, Mapk3, Mapre1, Mastl, Mau2, Mcm10, Mcm2, Mcm3, Mcm4, Mcm5, Mcm6, Mcm7, Mcm8, Mdc1, Mdm2, Mdm4, Mis12, Mis18a, Mis18bp1, Mnat1, Mre11a, Myc, Mzt1, Mzt2, Nbn, Ncapd2, Ncapd3, Ncapg, Ncapg2, Ncaph, Ncaph2, Ncf2, Nckap1l, Ndc1, Ndc80, Nde1, Ndel1, Nedd1, Nek2, Nek6, Nek7, Nek9, Ninl, Nipbl, Npc2, Nrros, Nsd2, Nsl1, Nudc, Nuf2, Numa1, Nup107, Nup133, Nup153, Nup155, Nup160, Nup188, Nup205, Nup210, Nup214, Nup35, Nup37, Nup43, Nup50, Nup54, Nup62, Nup85, Nup88, Nup93, Nup98, Nupl1, Nupl2, Odf2, Ofd1, Oip5, Optn, Orc1, Orc2, Orc3, Orc4, Orc5, Orc6, Pafah1b1, Pcm1, Pcna, Pcnt, Pds5a, Pds5b, Phf20, Phf8, Phlda1, Pias4, Pkmyt1, Plek, Plk1, Plk4, Pmf1, Pola1, Pola2, Pold1, Pold1it, Pold2, Pold3, Pold4, Pole, Pole2, Pole3, Pole4, Pom121, Ppme1, Ppp1cb, Ppp1cc, Ppp1r12a, Ppp1r12b, Ppp1r18, Ppp2ca, Ppp2cb, Ppp2r1a, Ppp2r1b, Ppp2r2a, Ppp2r3d, Ppp2r5a, Ppp2r5b, Ppp2r5c, Ppp2r5d, Ppp2r5e, Prdm9, Prim1, Prim2, Prkar2b, Prkca, Prkcb, Prkdc, Ptk6, Pttg1, Pycard, Rab1a, Rab1b, Rab2a, Rab8a, Rad1, Rad17, Rad21, Rad9a, Rad9b, Rae1, Ranbp2, Rangap1, Rb1, Rbbp4, Rbbp7, Rbbp8, Rbl1, Rbl2, Rbm47, Rbx1, Rcc2, Rfc1, Rfc2, Rfc3, Rfc4, Rfc5, Rgs1, Rhno1, Rmi1, Rmi2, Rnase6, Rnaseh1, Rnaseh2a, Rnaseh2b, Rnaseh2c, Rnf168, Rnf8, Rpa1, Rpa2, Rpa3, Rps27, Rps27a, Rps27rt, Rps6ka1, Runx3, Ruvbl1, Samsn1, Sdccag8, Sec13, Seh1l, Sfi1, Sfn, Sft2d2, Sgo1, Sgo2a, Sgo2b, Sh2b3, Ska1, Ska2, Skp1a, Skp2, Slc1a5, Slc7a7, Smad2, Smad3, Smad4, Smc1a, Smc1b, Smc2, Smc3, Smc4, Spc24, Spc25, Spdl1, Spp1, SrcRouss, Ssbp1, Ssna1, Stag1, Stag2, Stat5a, Sumo1, Taok1, Tcirg1, Tert, Tfdp1, Tfdp2, Tgfb1, Tgfb2, Tgfb3, Tgfbr1, Tmem106a, Tnfrsf1b, Top3a, Topbp1, Tpr, Tpx2, Trp53, Trp53bp1, Ttk, Tuba1a, Tuba4a, Tubb4a, Tubb4b, Tubb5, Tubg1, Tubg2, Tubgcp2, Tubgcp3, Tubgcp4, Tubgcp5, Tubgcp6, Tyrobp, Uba52, Ubb, Ube2c, Ube2d1, Ube2e1, Ube2v2, Uimc1, Vrk1, Vrk2, Wapl, Wee1, Wee2, Wrn, Xpo1, Ywha, Ywhab, Ywhae, Ywhag, Ywhah, Ywhaq, Ywhaz, Zbtb17, Zfp36l2, Zfp385a, Zw10, Zwilch, Zwint

**1386 Metabolism Genes** (KEGG). 1700029P11Rik, 1700080E11Rik, 2810007J24Rik, 9130409I23Rik, A2m, Aaas, Aadat, Aanat, Aasdhppt, Aass, Abca1, Abcb11, Abcb1a, Abcc1, Abcc3, Abcc5, Abcc8, Abcd1, Abcg1, Abcg2, Abcg5, Abcg8, Abhd5, Acaa1a, Acaa1b, Acaca, Acacb, Acad8, Acadl, Acadm, Acads, Acadsb, Acadvl, Acat1, Acer1, Acer2, Acer3, Ache, Acly, Acmsd, Aco2, Acot8, Acox1, Acox2, Acox3, Acp5, Acsl1, Acsl4, Acsl5, Acsl6, Acsm1, Acsm2, Acss1, Acss2, Ada, Adal, Adc, Adcy1, Adcy2, Adcy3, Adcy4, Adcy5, Adcy6, Adcy7, Adcy8, Adcy9, Adh1, Adh4, Adh6a, Adh6b, Adh7, Adhfe1, Adi1, Adk, Adra2c, Adsl, Adss, Adssl1, Afmid, Agl, Agpat1, Agpat2, Agpat3, Agpat4, Agpat5, Agpat6, Agpat9, Agps, Agxt, Agxt2, Ahcy, Ak1, Ak2, Ak5, Akap5, Akr1b3, Akr1c12, Akr1c13, Akr1c14, Akr1c18, Akr1c19, Akr1c20, Akr1c21, Akr1c6, Akr1cl, Akr1d1, Akt1, Alad, Alas1, Alas2, Alb, Aldh18a1, Aldh1a1, Aldh1a7, Aldh2, Aldh4a1, Aldh6a1, Aldh7a1, Aldh9a1, Aldoa, Aldoart1, Aldoart2, Aldob, Aldoc, Alox12, Alox12b, Alox15, Alox5, Alox5ap, Alox8, Amacr, Amd1, Amd2, Amdhd1, Amn, Ampd1, Ampd2, Ampd3, Amy1, Amy2a1, Amy2a2, Amy2a3, Amy2a4, Amy2a5, Aox1, Apip, Apoa1, Apoa2, Apoa4, Apoa5, Apob, Apoc3, Apoc4, Apoe, Aprt, Aqp1, Arf1, Arf3, Arg1, Arg2, Arsa, Arsb, Arsi, Arsj, Arsk, Asah1, Asah2, Asl, Asns, Ass1, Atic, Atp5a1, Atp5b, Atp5c1, Atp5d, Atp5e, Atp5f1, Atp5g1, Atp5h, Atp5j, Atp5j2, Atp5k, Atp5l, Atp5o, ATP6, ATP8, Auh, Azin1, B3galt6, B3gat1, B3gat2, B3gat3, B3gnt1, B3gnt2, B3gnt3, B3gnt4, B3gnt7, B4galt1, B4galt2, B4galt3, B4galt4, B4galt5, B4galt6, B4galt7, Baat, Bbox1, Bcan, Bcat1, Bcat2, Bckdha, Bckdhb, Bdh1, Bgn, Bhmt, Blvra, Blvrb, Bmp1, Bpnt1, Bsg, Btd, Cacna1a, Cacna1c, Cacna1d, Cacna2d2, Cacnb2, Cacnb3, Cad, Calm1, Calm2, Calm3, Car1, Car12, Car13, Car14, Car2, Car3, Car4, Car5a, Car5b, Car6, Car7, Car9, Carm1, Cat, Cav1, Cbr1, Cbs, Ccbl1, Ccbl2, Cd320, Cd44, Cda, Cdipt, Cdo1, Cds1, Cds2, Cel, Cept1, Cerk, Cers1, Cers2, Cers3, Cers4, Cers5, Cers6, Cga, Ch25h, Chat, Chd9, Chka, Chkb, Chpf, Chpf2, Chpt1, Chrm3, Chst1, Chst11, Chst12, Chst13, Chst14, Chst15, Chst2, Chst3, Chst5, Chst7, Chst9, Chsy1, Chsy3, Ciapin1, Ckb, Ckm, Ckmt1, Ckmt2, Clps, Cmpk1, Cndp2, Coasy, Col4a3bp, Comt, Coq2, Coq3, Coq5, Coq6, Coq7, COX1, Cox10, Cox15, COX2, COX3, Cox4i1, Cox5a, Cox5b, Cox6a1, Cox6b1, Cox6c, Cox7a2l, Cox7b, Cox7c, Cox8a, Cpox, Cps1, Cpt1a, Cpt1b, Cpt2, Crat, Crebbp, Crls1, Crot, Cs, Csad, Csgalnact1, Csgalnact2, Csl, Csnk1g2, Cspg4, Cspg5, Cth, Ctps, Ctps2, Ctsa, Cubn, Cyb5, Cyb5r3, Cyc1, Cycs, Cyp11a1, Cyp11b1, Cyp11b2, Cyp17a1, Cyp19a1, Cyp1a1, Cyp1a2, Cyp1b1, Cyp24a1, Cyp26a1, Cyp26b1, Cyp26c1, Cyp27a1, Cyp27b1, Cyp2a12, Cyp2a22, Cyp2a4, Cyp2a5, Cyp2b10, Cyp2b13, Cyp2b19, Cyp2b23, Cyp2b9, Cyp2c29, Cyp2c37, Cyp2c38, Cyp2c39, Cyp2c40, Cyp2c50, Cyp2c54, Cyp2c55, Cyp2c65, Cyp2c66, Cyp2c67, Cyp2c69, Cyp2d10, Cyp2d11, Cyp2d12, Cyp2d22, Cyp2d26, Cyp2d40, Cyp2d9, Cyp2e1, Cyp2f2, Cyp2j11, Cyp2j12, Cyp2j13, Cyp2j5, Cyp2j6, Cyp2j8, Cyp2j9, Cyp2r1, Cyp2s1, Cyp2u1, Cyp2w1, Cyp39a1, Cyp3a11, Cyp3a13, Cyp3a16, Cyp3a25, Cyp3a41a, Cyp3a41b, Cyp3a44, Cyp3a57, Cyp3a59, Cyp46a1, Cyp4a10, Cyp4a12a, Cyp4a12b, Cyp4a14, Cyp4a31, Cyp4a32, Cyp4b1, Cyp4f14, Cyp4f15, Cyp4f18, Cyp4f40, Cyp51, Cyp7a1, Cyp7b1, Cyp8b1, D2hgdh, Dao, Dbh, Dbt, Dck, Dcn, Dctd, Ddc, Decr1, Degs1, Degs2, Dgat1, Dgat2, Dguok, Dhcr24, Dhcr7, Dhfr, Dhodh, Dio1, Dio2, Dlat, Dld, Dlst, Dnm2, Dpep1, Dpep2, Dpyd, Dpys, Dse, Dsel, Dtymk, Dut, Ebp, Echs1, Eci1, Elovl1, Elovl2, Elovl3, Elovl4, Elovl5, Elovl6, Elovl7, Eno1, Eno2, Eno3, Enoph1, Enpp1, Enpp7, Ephx2, Epm2a, Ept1, Etfa, Etfb, Etfdh, Ethe1, Etnk1, Etnk2, Ext1, Ext2, Fabp1, Fabp4, Fabp6, Fads1, Fads2, Fah, Fam213b, Far1, Far2, Fasn, Fbp1, Fbp2, Fdft1, Fdps, Fdx1, Fdx1l, Fdxr, Fech, Ffar1, Fh1, Fig4, Flad1, Fmo1, Fmo2, Fmo3, Fmod, Fpgs, Ftcd, Fxn, G6pc, G6pc2, G6pc3, G6pd2, G6pdx, Gal3st1, Galc, Gale, Galk1, Galt, Gamt, Gapdh, Gapdhs, Gart, Gatm, Gba, Gba2, Gbe1, Gc, Gcdh, Gcg, Gcgr, Gch1, Gchfr, Gck, Gckr, Gclc, Gclm, Gda, Ggct, Ggps1, Ggt1, Ggt5, Gif, Gla, Glb1, Glce, Glp1r, Glrx, Gls, Gls2, Glud1, Glul, Glyat, Gm10053, Gm10681, Gm11273, Gm13342, Gm14680, Gm15776, Gm20899, Gm21464, Gm2962, Gm2a, Gm4353, Gm4356, Gm4450, Gm5506, Gm7293, Gm9769, Gmpr, Gmpr2, Gmps, Gna11, Gna14, Gna15, Gnai1, Gnai2, Gnaq, Gnas, Gnb1, Gnb2, Gnb3, Gnb4, Gnb5, Gng10, Gng11, Gng12, Gng13, Gng2, Gng3, Gng4, Gng5, Gng7, Gng8, Gngt1, Gngt2, Gnpat, Gns, Got1, Got2, Gpam, Gpat2, Gpc1, Gpc2, Gpc3, Gpc4, Gpc5, Gpc6, Gpcpd1, Gpd1, Gpd1l, Gpd2, Gphn, Gpi1, Gpt, Gpt2, Gpx1, Gpx2, Gpx4, Grhpr, Gsr, Gss, Gsta3, Gstm1, Gstm2, Gstm3, Gstm4, Gstm5, Gstm6, Gstm7, Gsto1, Gsto2, Gstp1, Gstp2, Gstt1, Gstt2, Gstt3, Gstz1, Guk1, Gusb, Gxylt1, Gyg, Gyk, Gykl1, Gys1, Gys2, Haao, Hacl1, Hadh, Hadha, Hadhb, Hal, Hao1, Hba-a1, Hbb-bh2, Hbb-bs, Hbb-bt, Hdac3, Hdc, Helz2, Hexa, Hexb, Hgd, Hibadh, Hibch, Hk1, Hk2, Hk3, Hlcs, Hmbs, Hmgcl, Hmgcr, Hmgcs1, Hmgcs2, Hmmr, Hmox1, Hmox2, Hpd, Hpgd, Hpgds, Hprt, Hpse, Hpse2, Hs2st1, Hs3st1, Hs3st2, Hs3st3a1, Hs3st3b1, Hs3st5, Hs3st6, Hs6st1, Hs6st2, Hsd11b1, Hsd17b1, Hsd17b10, Hsd17b12, Hsd17b3, Hsd17b4, Hsd17b7, Hsd3b1, Hsd3b2, Hsd3b3, Hsd3b4, Hsd3b5, Hsd3b6, Hsd3b7, Hsp90aa1, Hspg2, Hyal1, Hyal2, Idh1, Idh2, Idh3a, Idh3b, Idh3g, Idi1, Idi2, Ido1, Ido2, Ids, Idua, Impa1, Impa2, Impdh1, Impdh2, Inpp1, Inpp4a, Inpp4b, Inpp5a, Inpp5b, Inpp5d, Inpp5e, Inpp5j, Inpp5k, Inppl1, Ip6k1, Ip6k2, Ip6k3, Ipmk, Ippk, Iqgap1, Iscu, Isyna1, Itpa, Itpk1, Itpka, Itpkb, Itpkc, Itpr1, Itpr2, Itpr3, Ivd, Iyd, Jmjd7, Kcnj11, Kdsr, Kera, Khk, Kmo, Kpnb1, Kynu, L2hgdh, Lbr, Lcat, Lclat1, Lct, Ldha, Ldhb, Ldlr, Ldlrap1, Lhb, Lipc, Lipe, Lmbrd1, LOC100046079, LOC100048119, LOC100048410, Lpcat1, Lpcat2, Lpcat3, Lpcat4, Lpgat1, Lpin1, Lpin2, Lpin3, Lpl, Lrp2, Lss, Lta4h, Ltc4s, Lum, Lypla1, Lyrm4, Lyve1, Maoa, Maob, Mapkapk2, Marcks, Mat1a, Mat2a, Mat2b, Mboat1, Mboat2, Mboat7, Mbtps1, Mbtps2, Mccc1, Mccc2, Mcee, Mdh1, Mdh2, Med1, Mgam, Mgll, Mgst1, Mgst2, Mgst3, Minpp1, Mlx, Mlxipl, Mlycd, Mmaa, Mmab, Mmachc, Mmadhc, Mocos, Mocs1, Mocs2, Mocs3, Mpc1, Mpc2, Mri1, Mtap, Mthfd1, Mthfr, Mtmr1, Mtmr14, Mtmr2, Mtmr3, Mtmr4, Mtmr6, Mtmr7, Mtr, Mtrr, Mttp, Mut, Mvd, Mvk, Nadk, Nadsyn1, Naglu, Nags, Nampt, Naprt1, Nat1, Nat2, Nat3, Ncan, Ncoa1, Ncoa2, Ncoa6, Ncor1, Ncor2, ND4, ND6, Ndor1, Ndst1, Ndst2, Ndufa1, Ndufa10, Ndufa11, Ndufa12, Ndufa13, Ndufa2, Ndufa3, Ndufa4, Ndufa5, Ndufa6, Ndufa7, Ndufa8, Ndufa9, Ndufab1, Ndufb10, Ndufb11, Ndufb2, Ndufb3, Ndufb4, Ndufb5, Ndufb6, Ndufb7, Ndufb8, Ndufb9, Ndufc1, Ndufc2, Ndufs1, Ndufs2, Ndufs3, Ndufs4, Ndufs5, Ndufs6, Ndufs7, Ndufs8, Ndufv1, Ndufv2, Ndufv3, Neu1, Neu2, Neu3, Neu4, Nfs1, Nfya, Nfyb, Nfyc, Nme1, Nme2, Nme4, Nmnat1, Nmnat2, Nmnat3, Nnmt, Nnt, Nos3, Nosip, Nostrin, Nqo1, Nsdhl, Nt5c, Nt5c1a, Nt5c1b, Nt5c2, Nt5c3, Nt5e, Nt5m, Nudt1, Nudt10, Nudt11, Nudt15, Nudt16, Nudt18, Nudt3, Nudt4, Nudt5, Nudt9, Nup107, Nup133, Nup153, Nup155, Nup160, Nup188, Nup205, Nup210, Nup214, Nup35, Nup37, Nup43, Nup50, Nup54, Nup62, Nup85, Nup88, Nup93, Nup98, Nupl1, Nupl2, Oat, Oaz1, Oaz2, Oaz3, Ocrl, Odc1, Ogdh, Ogn, Omd, Osbp, Otc, Oxct1, P4hb, Pah, Paics, Pank1, Pank2, Paox, Papss1, Papss2, Pcbd1, Pcca, Pccb, Pck1, Pck2, Pcx, Pcyt1a, Pcyt1b, Pcyt2, Pdha1, Pdhb, Pdhx, Pdk1, Pdk2, Pdk3, Pdk4, Pdp1, Pdp2, Pdpr, Pdss1, Pdss2, Pdxk, Pemt, Pfas, Pfkfb1, Pfkfb2, Pfkfb3, Pfkfb4, Pfkl, Pfkm, Pfkp, Pgam1, Pgam2, Pgd, Pgk1, Pgls, Pgm1, Pgm2, Pgs1, Phgdh, Phka1, Phka2, Phkb, Phkg1, Phkg2, Phospho1, Phyh, Pi4k2a, Pi4k2b, Pi4ka, Pi4kb, Pik3c2a, Pik3c2b, Pik3c3, Pik3ca, Pik3cb, Pik3cd, Pik3cg, Pik3r1, Pik3r2, Pik3r3, Pik3r4, Pik3r5, Pikfyve, Pip4k2a, Pip4k2b, Pip5k1a, Pip5k1b, Pip5k1c, Pisd, Pitpnb, Pklr, Pkm, Pla2g10, Pla2g12a, Pla2g16, Pla2g1b, Pla2g2d, Pla2g2e, Pla2g2f, Pla2g3, Pla2g4a, Pla2g4b, Pla2g4c, Pla2g4d, Pla2g4f, Pla2g5, Pla2g6, Plb1, Plbd1, Plcb1, Plcb2, Plcb3, Plcb4, Plcd1, Plcd3, Plce1, Plcg1, Plcg2, Plch1, Plch2, Plcz1, Pld1, Pld2, Pld3, Pld4, Pld6, Plg, Plin1, Pltp, Pmvk, Pnlip, Pnmt, Pnp, Pnp2, Pnpla2, Pnpla3, Pnpla8, Pnpo, Pom121, Pomc, Ppap2a, Ppap2b, Ppap2c, Ppara, Ppat, Ppcdc, Ppcs, Ppip5k1, Ppip5k2, Ppm1l, Ppox, Ppp1ca, Ppp1cb,Ppp1cc, Ppp1r3c, Ppp2ca, Ppp2cb, Ppp2r1a, Ppp2r1b, Ppp2r5d, Prelp, Prkaa2, Prkab2, Prkaca, Prkacb, Prkag2, Prkar1a, Prkar1b, Prkar2a, Prkar2b, Prkca, Prkd1, Prkg2, Prodh, Prps1, Prps1l3, Psap, Psat1, Psph, Ptdss1, Ptdss2, Pten, Ptgds, Ptges, Ptges3, Ptgis, Ptgr1, Ptgr2, Ptgs1, Ptgs2, Ptpmt1, Pts, Pycr1, Pycr2, Pygb, Pygl, Pygm, Qdpr, Qprt, Rae1, Ran, Ranbp2, Rap1a, Rap1b, Rapgef3, Rapgef4, Rfk, Rhag, Rpe, Rpia, Rrm1, Rrm2, Rrm2b, Rxra, Sacm1l, Sar1b, Sat1, Sc4mol, Sc5d, Scap, Scarb1, Scp2, Sdc1, Sdc2, Sdc3, Sdc4, Sdha, Sdhb, Sdhc, Sdhd, Seh1l, Sgms1, Sgms2, Sgpl1, Sgpp1, Sgpp2, Sgsh, Shmt1, Sin3a, Sin3b, Sis, Slc10a1, Slc10a2, Slc16a1, Slc16a3, Slc16a8, Slc19a1, Slc19a2, Slc19a3, Slc22a1, Slc22a2, Slc22a3, Slc23a1, Slc23a2, Slc25a1, Slc25a10, Slc25a11, Slc25a12, Slc25a13, Slc25a15, Slc25a16, Slc25a17, Slc25a2, Slc25a20, Slc25a21, Slc25a28, Slc25a32, Slc25a37, Slc25a4, Slc25a5, Slc26a1, Slc26a2, Slc27a2, Slc27a5, Slc2a1, Slc2a2, Slc2a3, Slc2a4, Slc2a5, Slc35b3, Slc35d1, Slc35d2, Slc44a1, Slc44a2, Slc44a3, Slc44a4, Slc44a5, Slc46a1, Slc4a1, Slc52a3, Slc5a1, Slc5a5, Slc5a6, Slc6a8, Slc9a1, Slco1a1, Slco1a4, Slco1a6, Slco1b2, Smarcd3, Smox, Smpd1, Smpd2, Smpd3, Smpd4, Sms, Sphk1, Sphk2, Spr, Sptlc1, Sptlc2, Sptlc3, Sqle, Sqrdl, Srd5a1, Srd5a3, Srebf1, Srebf2, Srm, St3gal1, St3gal2, St3gal3, St3gal4, St3gal6, Stab2, Star, Stard5, Stk11, Sucla2, Suclg1, Suclg2, Sult1a1, Sult1b1, Sult1c2, Sult1e1, Sult2a1, Sult2a2, Sult2a3, Sult2a4, Sult2a6, Sult2b1, Sult4a1, Sumf1, Sumf2, Suox, Synj1, Synj2, Taldo1, Tat, Taz, Tbl1x, Tbl1xr1, Tbxas1, Tcn2, Tdo2, Tecr, Tgs1, Th, Thtpa, Tk1, Tk2, Tkt, Tm7sf2, Tmlhe, Tph1, Tph2, Tpi1, Tpk1, Tpmt, Tpo, Tpr, Tpte, Treh, Tshb, Tst, Txn1, Txnrd1, Tymp, Tyms, Uck1, Uck2, Ucp1, Ucp2, Ucp3, Ugcg, Ugdh, Ugp2, Ugt1a1, Ugt1a2, Ugt1a5, Ugt1a6a, Umps, Upb1, Upp1, Upp2, Uqcr10, Uqcr11, Uqcrb, Uqcrc1, Uqcrc2, Uqcrfs1, Uqcrh, Uqcrq, Uroc1, Urod, Uros, Ust, Vac14, Vapa, Vapb, Vcan, Wasl, Xdh, Zdhhc21.

**32 Cholesterol Biosynthesis Genes** (nonredundant list combining cholesterol biosynthesis from Reactome, MouseCyc and WikiPathways). Cyp51, Dhcr24, Dhcr7, Ebp, Elovl6, Fdft1, Fdps, Ggps1, Hmgcr, Hmgcs1, Hsd17b7, Hspg2, Idi1, Idi2, Lbr, Ldlr, Lss, Msmo1, Mttp, Mvd, Mvk, Nsdhl, P4hb, Pmvk, Ppp1cb, Ran, Sc4mol, Sc5d, Sdc1, Sqle, Srebf2, Tm7sf2.

**Figure 3C Gene Sets.**

**171 Translation Genes** (Reactome) (no pseudogenes). Ddost, Eef1a1, Eef1b2, Eef1d, Eef1g, Eef2, Eif1a, Eif1ad, Eif1ax, Eif2a, Eif2b1, Eif2b2, Eif2b3, Eif2b4, Eif2b5, Eif2s1, Eif2s2, Eif2s3x, Eif2s3y, Eif3a, Eif3b, Eif3c, Eif3d, Eif3e, Eif3f, Eif3g, Eif3h, Eif3i, Eif3j1, Eif3j2, Eif3k, Eif4a1, Eif4a2, Eif4b, Eif4e, Eif4ebp1, Eif4g1, Eif4h, Eif5, Eif5a, Eif5b, Etf1, Fau, Gm10029, Gm10071, Gm10177, Gm10237, Gm11575, Gm11810, Gm12816, Gm13141, Gm13826, Gm13841, Gm14279, Gm14438, Gm15427, Gm4705, Gm5481, Gm5621, Gm6109, Gm6251, Gm6404, Gm6570, Gm6887, Gm8618, Gspt2, LOC100045367, LOC100046223, LOC100047658, LOC100504872, LOC100504988, LOC100862587, LOC100862595, LOC101055915, LOC677113, Pabpc1, Rpl10, Rpl10a, Rpl11, Rpl12, Rpl13, Rpl13a, Rpl14, Rpl17, Rpl18, Rpl18a, Rpl19, Rpl22, Rpl23, Rpl23a, Rpl24, Rpl26, Rpl27, Rpl27a, Rpl29, Rpl3, Rpl30, Rpl32, Rpl34, Rpl35, Rpl35a, Rpl37, Rpl37a, Rpl38, Rpl39, Rpl3l, Rpl4, Rpl5, Rpl6, Rpl7, Rpl8, Rpl9, Rplp0, Rplp1, Rplp2, Rpn1, Rpn2, Rps10, Rps11, Rps12, Rps13, Rps14, Rps15, Rps15a, Rps16, Rps17, Rps18, Rps19, Rps2, Rps20, Rps21, Rps23, Rps24, Rps25, Rps27, Rps27a, Rps28, Rps29, Rps3, Rps3a1, Rps4x, Rps5, Rps6, Rps7, Rps8, Rps9, Rpsa, Sec11a, Sec11c, Sec61a1, Sec61a2, Sec61b, Sec61g, Spcs1, Spcs2, Spcs3, Srp14, Srp19, Srp54a, Srp54b, Srp54c, Srp68, Srp72, Srp9, Srpr, Srprb, Ssr1, Ssr2, Ssr3, Ssr4, Tram1, Uba52, Ubb, Ubc, Zbtb11.

**130 Amino Acid Synthesis Genes** (nonredundant compilation of KEGG pathways for phenylalanine, arginine & proline, cysteine & methionine, and glycine, threonine & serine pathways). 1700055N04Rik, 4930438A08Rik, Acy1, Adc, Adi1, Agmat, Agxt, Agxt2, Ahcy, Ahcyl1, Ahcyl2, Alas1, Alas2, Aldh18a1, Aldh1a3, Aldh1b1, Aldh2, Aldh3a1, Aldh3a2, Aldh3b1Aldh3b2, Aldh4a1, Aldh7a1, Aldh9a1, Amd1, Amt, Aoc1, Aoc2, Aoc3, Apip, Arg1, Arg2, Asl, Ass1, Bhmt, Bpgm, Carns1, Cbs, Cdo1, Chdh, Ckb, Ckm, Ckmt1, Ckmt2, Cndp1, Cndp2, Cps1, Cth, Dao, Ddc, Dld, Dmgdh, Dnmt1, Dnmt3a, Dnmt3b, Enoph1, Gamt, Gatm, Gcat, Gldc, Gls, Gls2, Glud1, Glul, Glyat, Glyctk, Gm14680, Gm4952, Gnmt, Got1, Got2, Grhpr, Hpd, Il4i1, Lao1, Lap3, Ldha, Ldhal6b, Ldhb, Ldhc, LOC100047252, LOC641049, Maoa, Maob, Mat1a, Mat2a, Mat2b, Mif, Mpst, Mri1, Mtap, Mtr, Nags, Nos1, Nos2, Nos3, Nup62-il4i1, Oat, Odc1, Otc, P4ha1, P4ha2, P4ha3, Pah, Pgam1, Pgam2, Phgdh, Pipox, Prdx6, Prodh, Prodh2, Psat1, Psph, Pycr1, Pycr2, Pycrl, Sardh, Sat1, Sat2, Sds, set, Shmt1, Shmt2, Sms, Srm, Srr, Tat, Tdh, Tha1, Tst32.

**32 Genes of Amino Acid Transport Across the Plasmalemma** (Reactome). AU018091, Slc16a10, Slc1a4, Slc1a5, Slc36a1, Slc36a2, Slc38a1, Slc38a2, Slc38a4, Slc38a5, Slc3a1, Slc3a2, Slc43a1, Slc43a2, Slc6a12, Slc6a14, Slc6a15, Slc6a18, Slc6a19, Slc6a20a, Slc6a20b, Slc6a6, Slc7a1, Slc7a10, Slc7a11, Slc7a2, Slc7a3, Slc7a5, Slc7a6, Slc7a7, Slc7a8, Slc7a9.

**41 tRNA Aminoacylation Genes** (Reactome). Aars, Aars2, Aimp2, Cars, Cars2, Dars, Dars2, Ears2, Eef1e1, Eprs, Fars2, Farsa, Farsb, Gars, Hars, Hars2, Iars, Iars2, Kars, Lars, Lars2, Mars, Mars2, Nars, Nars2, Pars2, Ppa1, Ppa2, Qars, Rars, Rars2, Sars, Sars2, Tars, Tars2, Vars, Vars2, Wars, Wars2, Yars, Yars2.

**147 Ribosome Biogenesis Genes (RiBi)** (Clauvin et al., *Oncogene* **33**:474, 2014). Aatf, Bms1, Bop1, Brix1, Bysl, Rmad1, Rbfa, Cdkn2a, Dcaf13, Ddx18, Ddx51, Ddx56, Dimt1, Dis3, Dkc1, Ebna1bp2, Ecm1, Eif2a, Eif4a3, Eif6, Emg1, ExosC1, Exosc10, ExosC2, Exosc3, Exosc4, Exosc5, Exosc6, ExosC7, Exosc8, Exosc9, Faf1, FbL, Fcf1, Frg1, Ftsj1, Ftsj2, Ftsj3, Gar1, Gemin4, Gnl2, Gnl3l, Gstcd, Gtpbp4, Ddx52, Heatr1, Imp3, Imp4, Isg20L2, Kri1, Krr1, Lrp1, Lsg1, Lsm2, Lsm4, Lsm5, Lsm6, Ltv1, Mak16, Mina, Mphosph10, Mrm1, Mrpl10, Mrpl11, Mtf1, Nhp2, Nhp2l1, Nip7, Nmd3, Nme1, Nob1, Noc4L, Nol9, Nolc1, Nop10, Nop14, Nop16, Nop2, Nop56, Nop58, Npm1, Npm3, Nsa2, Nup133, Nup188, Nup85, Pes1, Pno1, Pop1, Pop4, Pop5, Pop7, Pwp1, Pwp2, Rai1, Rcl1, RnaseL, Drosha, Rpf1, Rrp1, Rrp12, Rrp15, Rrp1b, Rrp8, Rrp9, Rrs1, Rsl24d1, Sbds, Sdad1, Sirt1, Ssu72, Surf6, Suv39h1, Tbl3, Tfb1m, Tgs1, Tom1, Tsr2, Urb2, Utp11L, Utp15, Utp20, Utp23, Utp6, Wbp11, Wdr12, Wdr36, Wdr55, Rrn3, Tcof1, Ddx21, Polr1a, Polr1b, Polr1c, Polr1d, Polr1e, Ubtf, Taf1a, Taf1b, Taf1c, Rnf8, Dnmt1, VhL, NcL, FbL, Npm1, NoLc1.

**73 Ribosomal Protein Genes** (73 of 79 are expressed in the pancreas, and excluding pseudogenes; Ribosomal Protein Gene Database). Rpl10, Rpl10a, Rpl11, Rpl12, Rpl13, Rpl13a, Rpl14, Rpl15, Rpl17, Rpl18, Rpl18a, Rpl19, Rpl21, Rpl22, Rpl23, Rpl23a, Rpl24, Rpl27, Rpl27a, Rpl28, Rpl29, Rpl3, Rpl31, Rpl32, Rpl34, Rpl35, Rpl35a, Rpl36, Rpl36a, Rpl37, Rpl37a, Rpl38, Rpl39, Rpl4, Rpl41, Rpl5, Rpl6, Rpl7, Rpl7a, Rpl8, Rpl9, Rplp0, Rplp1, Rps10, Rps11, Rps12, Rps13, Rps14, Rps15, Rps15a, Rps16, Rps17, Rps18, Rps19, Rps2, Rps20, Rps21, Rps23, Rps24, Rps25, Rps26, Rps28, Rps29, Rps3, Rps3a, Rps4x, Rps4y2, Rps5, Rps6, Rps7, Rps8, Rps9, Rpsa.

**36 Translation Initiation Factor Genes** (KEGG). Eif1a, Eif1ad, Eif1ax, Eif2a, Eif2b1, Eif2b2, Eif2b3, Eif2b4, Eif2b5, Eif2c2, Eif2s1, Eif2s2, Eif2s3x, Eif2s3y, Eif3a, Eif3b, Eif3c, Eif3d, Eif3e, Eif3f, Eif3g, Eif3h, Eif3i, Eif3j1, Eif3j2, Eif3k, Eif4a1, Eif4a2, Eif4b, Eif4e, Eif4ebp1, Eif4g1, Eif4h, Eif5, Eif5a, Eif5b.

**125 Protein Synthesis Regulator Genes** (Tcherkezian & Roux, *Genes Dev* **28**:357, 2014; plus Nucleolar Stress Response: A Nemeth et al., *Curr Opin Cell Biol* **52**:105, 2018; rDNA transcription: Bosio et al, *Transcription* **8**:254, 2017 and Russell et al., *Biochem Soc Symp* **73**:203, 2006; Kozak recognition: Hinnebusch, *Trends Biochem Sci* **32**:589, 2017; TISU proteins: Haimov & Dikstein, *Mol Cell Biol* **37**:e00150-17, 2017; 3’ ARE binding proteins: Barreau *Nucl Acids Res* **33**:7138, 2005.) Akt1, Akt1s1, Akt2, Cdk1, Cdk11b, Cit, Csnk1a1, Csnk1e, Csnk2a1, Ddx3x, Deptor, Eef2, Eef2k, Eif1, Eif1a, Eif1ad, Eif2a, Eif2ak4, Eif2c2, Eif3a, Eif3b, Eif3c, Eif3d, Eif3h, Eif4a1, Eif4a2, Eif4a3, Eif4B, Eif4e, Eif4Ebp1, Eif4Ebp2, Eif4Ebp3, Eif4g1, Eif4g2, Eif5, Eif5a, Gemin5, Grb2, Grk4, Gsk3b, HBXIP, Hnrnpu, Imp3, Imp4, Insr, Irs1, Irs2, Kdm4a, Kras, Lamtor1, Lamtor2, Lamtor3, Lamtor4, Larp1, Lats1, Lats2, Maf1, Mapk1, Mapk3, Mapkap1, Mast, Mknk1, Mknk2, Mlst8, Mphosph10, Msin1, mTOR, Ncbp1, Nf1, Pabpc4, Pabpn1, Pak2, Pdk1, Phlpp1, Phlpp2, Pim2, Ppm1g, Ppp1ca, Ppp1cc, Ppp1r15a, Ppp1r15b, Ppp2ca, Ppp2cb, Ppp2r1a, Ppp2r1b, Ppp2r2a, Ppp2r2d, Ppp2r5a, Ppp2r5c, Ppp2r5e, Ppp6c, Prkx, Prr5, Prr5L, Pten, Raptor, Ras, Rheb, Rictor, Rpa1, Rps10, Rps3, Rps6, Rps6ka1, Rps6ka2, Rps6ka3, Rps6ka4, Rps6ka5, Rps6ka6, Rps6kb1, Rps6kb2, Rptor, Rrn3, Sgk1, Sgk2, SgkNdr, Sh3bp4, Shc1, Sos1, Sos2, Stk38, Tbc1d7, Tbk1, Tsc1, Tsc2pp.

**35 Signal Recognition Particle and Protein Export Genes** (Reactome). Immp1L, Srp9P1, Immp2L, Oxa1L, Sec62, Sec63, Ddost, Rpsa, Sec11a, Sec11c, Sec61a1, Sec61a2, Sec61b, Sec61g, Spcs1, Spcs2, Spcs3, Srp14, Srp19, Srp54a, Srp54b, Srp54c, Srp68, Srp72, Srp9, Srpr, Srprb, Ssr1, Ssr2, Ssr3, Ssr4, Tram1, Uba52, Ubb, Ubc, Zbtb11.

**78 Asparagine N-glycosylation Genes** (Reactome). Alg1, Alg10b, Alg11, Alg12, Alg14, Alg2, Alg3, Alg5, Alg6, Alg8, Alg9, B4galt1, B4galt2, B4galt3, B4galt4, B4galt5, B4galt6, Calr, Canx, Dolk, Dolpp1, Dpagt1, Dpm1, Dpm2, Dpm3, Edem1, Edem2, Edem3, Fut8, Ganab, Gfpt1, Gfpt2, Gmppa, Gmppb, Gnpnat1, Lman1, Man1a, Man1a2, Man1b1, Man1c1, Man2a1, Man2a2, Manea, Mcfd2, Mgat1, Mgat2, Mgat3, Mgat4a, Mgat4b, Mgat4c, Mgat5, Mlec, Mogs, Mpi, Mvd, Pdia3, Pgm3, Pmm1, Pmm2, Preb, Prkcsh, Rft1, Sar1b, Sec13, Sec23a, Sec24a, Sec24b, Sec24c, Sec24d, Sec31a, St3gal4, St6gal1, St8sia2, St8sia3, St8sia6, Uap1, Uggt1, Uggt2.

**168 Protein Processing Genes** (KEGG). Amfr, Atf4, Atf6, Atf6b, Atxn3, Bag1, Bag2, Bak1, Bax, Bcap31, Bcl2, Calr, Canx, Capn1, Capn2, Casp12, Ckap4, Cryaa, Cryab, Cul1, Dad1, Ddit3, Ddost, Derl1, Derl2, Derl3, Dnaja1, Dnaja2, Dnajb1, Dnajb11, Dnajb12, Dnajb2, Dnajc1, Dnajc10, Dnajc3, Dnajc5, Dnajc5b, Dnajc5g, Edem1, Edem2, Edem3, Eif2ak1, Eif2ak2, Eif2ak3, Eif2ak4, Eif2s1, Erlec1, Ern1, Ero1l, Ero1lb, Erp29, Fbxo2, Fbxo6, Ganab, Gm10144, Gm10177, Gm11575, Gm15266, Gm9840, Herpud1, Hsp90aa1, Hsp90ab1, Hsp90b1, Hspa1a, Hspa1b, Hspa1l, Hspa2, Hspa4l, Hspa5, Hspa8, Hspbp1, Hsph1, Hyou1, Lman1, Lman1l, Lman2, Man1a, Man1a2, Man1b1, Man1c1, Map2k7, Map3k5, Mapk10, Mapk8, Mapk9, Mar6, Mbtps1, Mogs, Nfe2l2, Ngly1, Nploc4, Nsfl1c, Os9, P4hb, Park2, Pdia3, Pdia4, Pdia6, Plaa, Ppp1r15a, Preb, Prkcsh, Rad23a, Rad23b, Rbx1, Rnf185, Rnf5, Rpn1, Rpn2, Rrbp1, Sar1a, Sar1b, Sec13, Sec23a, Sec23b, Sec24a, Sec24b, Sec24c, Sec24d, Sec31a, Sec31b, Sec61a1, Sec61a2, Sec61b, Sec61g, Sec62, Sec63, Sel1l, Sil1, Skp1a, Ssr1, Ssr2, Ssr3, Ssr4, Stt3a, Stt3b, Stub1, Svip, Syvn1, Traf2, Tram1, Tusc3, Txndc5, Ube2d1, Ube2d2a, Ube2d3, Ube2e1, Ube2e2, Ube2e3, Ube2g1, Ube2g2, Ube2j1, Ube2j2, Ube4b, Ubqln1, Ubqln2, Ubqln3, Ubqln4, Ubqlnl, Ubxn6, Ufd1l, Uggt1, Uggt2, Vcp, Vimp, Wfs1, Xbp1, Yod1.

**Figure 4D Gene Sets.**

**63 DNA Replication Genes (**nonredundant list combining DNA replication from Reactome, KEGG and WikiPathways). Ccna1, Ccna2, Cdc45, Cdc6, Cdc7, Cdk2, Cdkn1a, Cdkn1b, Cdt1, Dbf4, Dna2, Fen1, Fzr1f, Gins1, Gins2, Gins4, Gmnn, Lig1, Mcm10, Mcm2, Mcm3, Mcm4, Mcm5, Mcm6, Mcm7, Mcm8, Orc1, Orc2, Orc3, Orc4, Orc5, Orc6, Pcna, Pola1, Pola2, Pold1, Pold2, Pold3, Pold4, Pole, Pole2, Pole3, Pole4, Prim1, Prim2, Rb1, Rfc1, Rfc2, Rfc3, Rfc4, Rfc5, Rnaseh1, Rnaseh2a, Rnaseh2b, Rnaseh2c, Rpa1, Rpa2, Rpa3, Rps27a, Ssbp1, Uba52, Ubb, Ubc.

**29 DNA Strand Elongation Genes** (Reactome). Cdc45, Dna2, Fen1, Gins1, Gins2, Gins4, Lig1l, Mcm2, Mcm3, Mcm4, Mcm5, Mcm6, Mcm7, Mcm8, Pcna, Pola1, Pola2, Pold1, Pold2, Pold3, Pold4, Prim1, Rfc1, Rfc2, Rfc3, Rfc4, Rfc5, Rpa1, Rpa2, Rpa3.

**49 Regulation of Replication Genes** (Reactome and literature survey). Ccna1, Ccna2, Cdc6, Cdk2, Cdkn1a, Cdkn1b, Cdt1, Fzr1, Gmnn, Mcm10, Mcm2, Mcm3, Mcm4, Mcm5, Mcm6, Mcm7, Mcm8, Orc1, Orc2, Orc3, Orc4, Orc5, Orc6, Rb1, Rps27a, Uba52, Ubb, Ubc, Cdc7, Cdc45, Dbf4, Jun , Jund, Myc, Tfdp1, Tfdp2, E2f1, E2f2, Trp53, Nfkb2, Mknk2, Relb, RbL1.

**41 p53 Signaling Pathway Genes** (KEGG). Apaf1, Atm, Atr, Bai1, Bax, Bbc3, Bid, Casp3, Casp8, Casp9, Ccnb1, Ccnb2, Ccnb3, Ccnd1, Ccnd2, Ccnd3, Ccne1, Ccne2, Ccng1, Ccng2, Cd82, Cdk1, Cdk2c, Cdk4, Cdk6, Cdkn1a, Cdkn2a, Chek1, Chek2, Cycs, Cyct, Ddb2, Ei24, Fas, Gadd45aa, Gadd45b, Gadd45g, Gm5593, Gtse1, Igf1, Igfbp3, LOC640611, Lrdd, Mdm2, Mdm4, Perp, Pmaip1, Ppm1d, Pten, Rchy1, Rfwd2, Rprm, Rrm2, Rrm2b, Serpinb5, Serpine1, Sesn1, Sesn2, Sesn3, Sfn, Shisa5, Siah1a, Siah1b, Steap3, Thbs1, Trp53, Trp73, Tsc2, Zmat3.

**335 Cell Cycle Genes** (Reactome). Aaas, Actr1a, Ahctf1, Ajuba, Akap9, Alms1, Anapc1, Anapc1, Anapc2, Anapc4, Anapc5, Anapc7, Ankle2, Apitd1, Arpp19, Aurka, Aurkb, Azi1, B9d2, Banf1, Birc5, Blzf1, Bora, Btrc, Bub1, Bub1b, Bub3, Casc5, Ccna1, Ccna2, Ccnb1, Ccnb2, Ccnd1, Ccne1, Ccne2, Ccnh, Ccp110, Cdc14a, Cdc16, Cdc20, Cdc23, Cdc25a, Cdc25b, Cdc25c, Cdc26, Cdc27, Cdc45, Cdc6, Cdc7, Cdca5, Cdca8, Cdk1, Cdk2, Cdk4, Cdk5rap2, Cdk6, Cdk7, Cdkn1a, Cdkn1b, Cdkn2a, Cdkn2b, Cdkn2c, Cdkn2d, Cdt1, Cenpa, Cenpc1, Cenpe, Cenpf, Cenph, Cenpi, Cenpj, Cenpk, Cenpl, Cenpm, Cenpn, Cenpo, Cenpp, Cenpq, Cenpt, Cep135, Cep152, Cep164, Cep192, Cep250, Cep290, Cep41, Cep57, Cep70, Cep72, Cep76, Cep78, Cetn2, Ckap5, Clasp1, Clasp2, Clip1, Csnk1d, Csnk1e, Csnk2a1, Csnk2a2, Csnk2b, Cul1, Dbf4, Dctn1, Dctn2, Dctn3, Dna2, Dsn1, Dync1h1, Dync1i2, Dynll1, Dyrk1a, E2f1, E2f2, E2f3, E2f4, E2f5, Emd, Ensa, Ercc6l, Esco1, Esco2, Espl1, Fbxo5, Fbxw11, Fen1, Fgfr1op, Foxm1, Fzr1, Gins1, Gins2, Gins4, Gm10093, Gm4353, Gm6531, Gm7020, Gmnn, Golga2, Gorasp1, Gorasp2, Gsk3b, Haus2, Hdac1, Hdac8, Hsp90aa1, Incenp, Itgb3bp, Kif18a, Kif20a, Kif23, Kif2a, Kif2b, Kif2c, Kntc1, Lig1, Lin37, Lin52, Lin54, Lin9, Lmna, Mad1l1, Mad2l1, Mapk1, Mapk3, Mapre1, Mastl, Mau2, Mcm10, Mcm2, Mcm3, Mcm4, Mcm5, Mcm6, Mcm7, Mcm8, Mis12, Mlf1ip, Mnat1, Ncapd2, Ncapg, Ncaph, Ndc80, Nde1, Ndel1, Nedd1, Nek2, Nek6, Nek9, Ninl, Nipbl, Nsl1, Nudc, Nuf2, Numa1, Nup107, Nup133, Nup153, Nup155, Nup160, Nup188, Nup205, Nup210, Nup214, Nup35, Nup37, Nup43, Nup50, Nup54, Nup62, Nup85, Nup88, Nup93, Nup98, Nupl, Nupl2, Odf2, Ofd1, Optn, Orc1, Orc2, Orc3, Orc4, Orc5, Orc6, Pafah1b1, Pcm1, Pcna, Pcnt, Pds5a, Pds5b, Pkmyt1, Plk1, Plk4, Pmf1, Pola1, Pola2, Pold1, Pold2, Pold3, Pold4, Pole, Pole2, Pom121, Ppp1cb, Ppp1cc, Ppp1r12a, Ppp1r12b, Ppp2ca, Ppp2cb, Ppp2r1a, Ppp2r1b, Ppp2r2a, Ppp2r2d, Ppp2r5a, Ppp2r5b, Ppp2r5c, Ppp2r5d, Ppp2r5e, Prim1, Prkaca, Prkar2b, Pttg1, Rab1, Rab2a, Rab8a, Rad21, Rae1, Ranbp2, Rangap1, Rb1, Rbbp4, Rbl1, Rbl2, Rcc2, Rfc1, Rfc2, Rfc3, Rfc4, Rfc5, Rpa1, Rpa2, Rpa3, Rps27, Rps27a, Sdccag8, Sec13, Seh1l, Sfi1, Sgol1, Sgol2, Ska1, Ska2, Skp1a, Skp2, Smc1a, Smc2, Smc3, Smc4, Spc24, Spc25, Spdl1, Ssna1, Stag1, Stag2, Taok1, Tfdp1, Tfdp2, Tpr, Tuba1a, Tuba4a, Tubb4a, Tubb5, Tubg1, Tubg2, Tubgcp2, Tubgcp3, Tubgcp4, Tubgcp5, Tubgcp6, Uba52, Ubb, Ubc, Ube2c, Ube2d1, Ube2e1, Uso1, Vrk1, Wapal, Wee1, Xpo1, Ywhae, Ywhag, Zw10, Zwilch, Zwint.

**73 Cell Cycle Checkpoint Genes** (Reactome). Anapc1, Anapc10, Anapc2, Anapc4, Anapc5, Anapc7, Atm, Atr, Atrip, Bub1b, Bub3, Ccnb1, Ccnb2, Ccne1, Ccne2, Cdc16, Cdc20, Cdc23, Cdc25a, Cdc25c, Cdc26, Cdc27, Cdc45, Cdc6, Cdc7, Cdk1, Cdk2, Cdkn1a, Cdkn1b, Chek1, Chek2, Clspn, Dbf4, Hus1, Mad1l1, Mad2l1, Mcm10, Mcm2, Mcm3, Mcm4, Mcm5, Mcm6, Mcm7, Mcm8, Mdm2, Orc1, Orc2, Orc3, Orc4, Orc5, Orc6, Pkmyt1, Rad1, Rad17, Rad9a, Rad9b, Rfc2, Rfc3, Rfc4, Rfc5, Rfwd2, Rpa1, Rpa2, Rpa3, Rps27a, Trp53, Uba52, Ubb, Ubc, Ube2c, Ube2d1, Ube2e1, Wee1.

**Figure 5A Metabolism pathway gene sets.**

**57 Arginine and proline metabolism genes** (KEGG). Acy1, Adc, Agmat, Aldh18a1, Aldh1b1, Aldh2, Aldh3a2, Aldh4a1, Aldh7a1, Aldh9a1, Amd1, Aoc1, Arg1, Arg2, Asl, Ass1, Carns1, Ckb, Ckm, Ckmt1, Ckmt2, Cndp1, Cndp2, Cps1, Dao, Gamt, Gatm, Gls, Gls2, Glud1, Glul, Gm14680, Got1, Got2, Lap3, LOC641049, Maoa, Maob, Nags, Nos1, Nos2, Nos3, Oat, Odc1, Otc, P4ha1, P4ha2, P4ha3, Prodh, Prodh2, Pycr1, Pycr2, Pycrl, Sat1, Sat2, Sms, Srm.

**39 Glycine, serine and threonine metabolism genes** (KEGG). Agxt, Agxt2, Alas1, Alas2, Aldh7a1, Amt, Aoc2, Aoc3, Bhmt, Bpgm, Cbs, Chdh, Cth, Dao, Dld, Dmgdh, Gamt, Gatm, Gcat, Gldc, Glyctk, Gnmt, Grhpr, LOC100047252**,** Maoa, Maob, Pgam1, Pgam2, Phgdh, Pipox, Psat1, Psph, Sardh, Sds, Shmt1, Shmt2, Srr, Tdh, Tha1.

**53 Branched chain amino acid catabolism genes** (KEGG). 4930438A08Rik, Abat, Acaa1a, Acaa1b, Acaa2, Acad8, Acadm, Acads, Acadsb, Acat1, Acat2, Aldh1b1, Aldh2, Aldh3a2, Aldh6a1, Aldh7a1, Aldh9a1, Aox1, Aox3, Aox3l1, Aox4, Auh, Bcat1, Bcat2, Bckdha, Bckdhb, Dbt, Dld, Echs1, Ehhadh, Hadh, Hadha, Hadhb, Hibadh, Hibch, Hmgcl, Hmgcs1, Hmgcs2, Hsd17b10, Il4i1, Ivd, Lao1, LOC101056567, Mccc1, Mccc2, Mcee, Mut, Nup62-il4i1, Oxct1, Oxct2a, Oxct2b, Pcca, Pccb.

**38 Cysteine and methionine biosynthesis genes** (KEGG). 4930438A08Rik, Adi1, Ahcy, Ahcyl1, Ahcyl2, Amd1, Apip, Bhmt, Cbs, Cdo1, Cth, Dnmt1, Dnmt3a, Dnmt3b, Enoph1, Gm14680, Got1, Got2, Il4i1, Lao1, Ldha, Ldhal6b, Ldhb, Ldhc, LOC641049, Mat1a, Mat2a, Mat2b, Mpst, Mri1, Mtap, Mtr, Nup62-il4i1, Sds, Sms, Srm, Tat, Tst.

**32 Amino acid transport protein genes** (Reactome; restricted to the 32 Slc genes expressed in mouse pancreas). AU018091, Slc16a10, Slc1a4, Slc1a5, Slc36a1, Slc36a2, Slc38a1, Slc38a2, Slc38a4, Slc38a5, Slc3a1, Slc3a2, Slc43a1, Slc43a2, Slc6a12, Slc6a14, Slc6a15, Slc6a18, Slc6a19, Slc6a20a, Slc6a20b, Slc6a6, Slc7a1, Slc7a10, Slc7a11, Slc7a2, Slc7a3, Slc7a5, Slc7a6, Slc7a7, Slc7a8, Slc7a9.

**136 Purine metabolism genes** (KEGG; excluding RNA and DNA polymerase subunit genes). 1700080E11Rik, 4933425L06Rik, Ada, Adcy1, Adcy10, Adcy2, Adcy3, Adcy4, Adcy5, Adcy6, Adcy7, Adcy8, Adcy9, Adk, Adprm, Adsl, Adss, Adssl1, Ak1, Ak2, Ak4, Ak5, Ak7, Allc, Ampd1, Ampd2, Ampd3, Aprt, Atic, Cant1, Dck, Dguok, Enpp1, Enpp3, Entpd1, Entpd2, Entpd3, Entpd4, Entpd5, Entpd6, Entpd8, Fhit, Gart, Gda, Gm15210, Gmpr, Gmpr2, Gmps, Gucy1a2, Gucy1a3, Gucy1b2, Gucy1b3, Gucy2c, Gucy2d, Gucy2e, Gucy2f, Guk1, Hddc3, Hprt, Impdh1, Impdh2, Itpa, Nme1, Nme2, Nme3, Nme4, Nme5, Nme6, Nme7, Npr1, Npr2, Nt5c, Nt5c1a, Nt5c1b, Nt5c2, Nt5c3, Nt5c3b, Nt5e, Nt5m, Ntpcr, Nudt16, Nudt2, Nudt5, Nudt9, Paics, Papss1, Papss2, Pde10a, Pde11a, Pde1a, Pde1b, Pde1c, Pde2a, Pde3a, Pde3b, Pde4a, Pde4b, Pde4c, Pde4d, Pde5a, Pde6a, Pde6b, Pde6c, Pde6d, Pde6g, Pde6h, Pde7a, Pde7b, Pde8a, Pde8b, Pde9a, Pfas, Pgm1, Pgm2, Pklr, Pkm, Pnp, Pnp2, Pnpt1, Ppat, Prps1, Prps1l1, Prps1l3, Prps2, Prune, Rrm1, Rrm2, Rrm2b, Twistnb, Uox, Urad, Urah, Xdh, Znrd1, Gm21685, LOC100862456.

**65 Pyrimidine metabolism genes (**KEGG; excluding RNA and DNA polymerase subunit genes). 4933425L06Rik, Ak3, Cad, Cant1, Cda, Cmpk1, Cmpk2, Ctps, Ctps2, Dck, Dctd, Dctpp1, Dhodh, Dpyd, Dpys, Dtymk, Dut, Entpd1, Entpd3, Entpd4, Entpd5, Entpd6, Entpd8, Gm21685, Itpa, LOC100862456, Nme1, Nme2, Nme3, Nme4, Nme5, Nme6, Nme7, Nt5c, Nt5c1a, Nt5c1b, Nt5c2, Nt5c3, Nt5c3b, Nt5e, Nt5m, Nudt2, Pnp, Pnp2, Pnpt1, Rrm1, Rrm2, Rrm2b, Tk1, Tk2, Twistnb, Txnrd1, Txnrd2, Txnrd3, Tymp, Tyms, Uck1, Uck2, Uckl1, Umps, Upb1, Upp1, Upp2, Uprt, Znrd1.

**32 Citrate cycle genes** (KEGG). 4933405O20Rik, Acly, Aco1, Aco2, Cs, Csl, Dlat, Dld, Dlst, Fh1, Idh1, Idh2, Idh3a, Idh3b, Idh3g, Mdh1, Mdh2, Ogdh, Ogdhl, Pck1, Pck2, Pcx, Pdha1, Pdha2, Pdhb, Sdha, Sdhb, Sdhc, Sdhd, Sucla2, Suclg1, Suclg2.

**56 Carbohydrate metabolism genes** (Reactome; 56 of 242 genes selected for being affected by at least one dTF cKO). Aaas, Abcc5, Aldob, Amy1, B3galt6, B3gat3, B3gnt1, B4galt1, B4galt4, Bgn, Calm2, Chst15, Dcn, G6pdx, Gale, Galk1, Gck, Glb1, Gpc1, Gpc4, Hexa, Hexb, Hk2, Hspg2, Hyal2, Ids, Khk, Lum, Lyve1, Naglu, Ndst1, Nup155, Nup210, Nup85, Nup93, Ogn, Pck2, Pcx, Pfkl, Pfkm, Pgd, Pgls, Pklr, Ppp2cb, Ppp2r5d, Sdc1, Sdc2, Sdc3, Slc25a10, Slc2a1, St3gal1, St3gal3, St3gal4, Taldo1, Tkt, Ugp2.

**32 Cholesterol Biosynthesis Genes** (nonredundant list combining cholesterol biosynthesis from Reactome, MouseCyc and WikiPathways). Cyp51, Dhcr24, Dhcr7, Ebp, Elovl6, Fdft1, Fdps, Ggps1, Hmgcr, Hmgcs1, Hsd17b7, Hspg2, Idi1, Idi2, Lbr, Ldlr, Lss, Msmo1, Mttp, Mvd, Mvk, Nsdhl, P4hb, Pmvk, Ppp1cb, Ran, Sc4mol, Sc5d, Sdc1, Sqle, Srebf2, Tm7sf2.

**25 Phosphoinositol and phosphoinositol phosphate metabolism genes** (MouseCyc). Calm2, Inpp1, Inpp5b, Inpp5e, Inppl1, Ip6k1, Ippk, Isyna1, Itpka, Itpkb, Mtmr1, Ocrl, Pi4k2b, Pik3c2a, Pik3c2b, Pik3c3, Pik3r1, Pik3r2, Pip5k1b, Plcb4, Plcd1, Plcd3, Plcg1, Plcg2, Synj2.

**142 Oxidative phosphorylation genes** (KEGG). Atp12a, Atp4a, Atp4b, Atp5a1, Atp5b, Atp5c1, Atp5d, Atp5e, Atp5f1, Atp5g1, Atp5g2, Atp5g3, Atp5h, Atp5j, Atp5j2, Atp5k, Atp5l, Atp5o, ATP6, Atp6ap1, Atp6v0a1, Atp6v0a2, Atp6v0a4, Atp6v0b, Atp6v0c, Atp6v0d1, Atp6v0d2, Atp6v0e, Atp6v0e2, Atp6v1a, Atp6v1b1, Atp6v1b2, Atp6v1c1, Atp6v1c2, Atp6v1d, Atp6v1e1, Atp6v1e2, Atp6v1f, Atp6v1g1, Atp6v1g2, Atp6v1g3, Atp6v1h, ATP8, COX1, Cox10, Cox11, Cox15, Cox17, COX2, COX3, Cox4i1, Cox4i2, Cox5a, Cox5b, Cox6a1, Cox6a2, Cox6b1, Cox6b2, Cox6c, Cox7a1, Cox7a2, Cox7a2l, Cox7b, Cox7b2, Cox7c, Cox8a, Cox8b, Cox8c, Cyc1, CYTB, Gm3244, Gm3873, Gm4943, Gm6415, Lhpp, LOC100046079, LOC100047429, LOC100048613, LOC100504968, LOC631040, LOC635087, LOC675851, ND1, ND2, ND3, ND4, ND4L, ND5, ND6, Ndufa1, Ndufa10, Ndufa11, Ndufa12, Ndufa13, Ndufa2, Ndufa3, Ndufa4, Ndufa4l2, Ndufa5, Ndufa6, Ndufa7, Ndufa8, Ndufa9, Ndufab1, Ndufb10, Ndufb11, Ndufb2, Ndufb3, Ndufb4, Ndufb5, Ndufb6, Ndufb7, Ndufb8, Ndufb9, Ndufc1, Ndufc2, Ndufs1, Ndufs2, Ndufs3, Ndufs4, Ndufs5, Ndufs6, Ndufs7, Ndufs8, Ndufv1, Ndufv2, Ndufv3, Ppa1, Ppa2, Sdha, Sdhb, Sdhc, Sdhd, Tcirg1, Uqcr10, Uqcr11, Uqcrb, Uqcrc1, Uqcrc2, Uqcrfs1, Uqcrh, Uqcrq.

**32 Folate metabolism genes** (KEGG Folate biosynthesis and One carbon by folate). Akp3, Aldh1l1, Aldh1l2, Alpi, Alpl, Alppl2, Amt, Atic, Dhfr, Dhfr, Fpgs, Ftcd, Gart, Gch1, Ggh, Gm2382, Mocs1, Mocs2, Mtfmt, Mthfd1, Mthfd1l, Mthfd2, Mthfd2l, Mthfr, Mthfs, Mtr, Pts, Qdpr, Shmt1, Shmt2, Spr, Tyms.

**8 Creatine metabolism genes** (Reactome). Ckb, Ckm, Ckmt1, Ckmt2, Gamt, Gatm, Gnmt, Slc6a8.

**Sources**

**KEGG:** kegg.jp; Kanehisa & Goto *Nucleic Acids Res* **28**:27 (2000). PMID: 10592173

**Reactome:** reactome.org; Fabregat et al. BMC Bioinformatics **18**:142 (2017). PMID:28150241.

**WikiPathways:** WikiPathways.org; Kelder et al. *PLoS One* **4**:e6447 (2009). PMID:19649250.

**MouseCYC**: Evsikov et al. *Genome Biol* **10**:R84. PMID:19682380 (2009)
