# Supplementary material for: Key transcriptional effectors of the pancreatic acinar phenotype and oncogenic transformation
Source: PLoS One. 2023 Oct 5;18(10):e0291512. doi: 10.1371/journal.pone.0291512 (PMC10553828; doi:10.1371/journal.pone.0291512)
Supplement: S4 Table — (PDF) [file pone.0291512.s012.pdf]

S4 Table. Fourteen stomach- or intestine-restricted genes affected by Ptf1a-cKO and indicative of loss of acinar cell-identity.

	Gene	ESTppm Pancreas	EST ppm Stomach	EST ppm Intestine	UniGene Tissues*	RNAseq fold-change: Ptf1a	Nr5a2	Foxa2	Gata4	Foxa2 + Gata4	Protein ID
1	Pga5	0	251	0	4	**637.0**	bd**	0.94	0.03	**2.71**	Pepsinogen 5
2	Chia	37	6202	633	9	**321.8**	bd	bd	bd	**53.2**	Chitinase
3	Gcnt3	0	409	161	3	**237.5**	bd	bd	bd	**54.5**	Glucosaminyl transferase 3
4	Gif	0	472	23	2	**763.0**	bd	bd	bd	bd	Gastric intrinsic factor
5	Gkn3	0	62	126	4	**427.7**	bd	0.81	0.07	**41.3**	Gastrokine 3
6	Kcnj15	0	(341)^	0	8	**119.1**	**7.1**	bd	bd	**2.8**	K+ inwardly-rectifying channel, subfamily J, member 15
7	Pgc	65	10201	0	5	**3.0**	bd	0.38	bd	**2.5**	Progastricsin: included eventhough expressed in pancreas
8	Ppp1r3a	0	314	0	4	**235.6**	bd	bd	bd	bd	Protein phosphatase 1 regulatory subunit 3A
9	Abcc2	0	0	11	5	**51.3**	bd	bd	bd	**10.5**	ATP-binding cassette, subfamily C, member 2
10	Ifi27l2b	9	94	92	4	**110.0**	bd	**2.53**	bd	**6.4**	Interferon-inducible protein 27 like 2B of inner mito membrane
11	Muc5ac	0	2141	0	1	**144.1**	bd	bd	bd	bd	Acidic Mucin 5ac
12	Vsig2	0	377	21	6	**4.2**	bd	**3.07**	0.37	**4.5**	V-set and immunoglobulin domain containing protein 2
13	Kcne2	9	125	0	5	**5.1**	bd	bd	bd	**6.1**	Potassium voltage-gated channel, Isk-related subfamily, gene 2
14	Mecom					**3.2**	bd	bd	bd	**2.2**	Mds1-Evi1/Prdm3 transcriptional regulator

Stomach- and/or intestine-enriched genes identified from the UniGene (NCBI) derived list of 23 genes total with 'stomach enriched expression'.
* Number of adult tissues/organs with ESTs out of 37 listed in UniGene (NCBI). Restricted means expression in <10 tissues.
** bd, below detection in the RNA-Seq analysis. Generally means that derepression, if it occurs, does not raise the level to detection in an RNAseq analysis.
^ No stomach ESTs in UniGene database. GNF Mouse GeneAtlas (GSE10246) distribution for Kcnj15 mRNA, 6 organs (pancreas, bd; stomach, 341; intestine, bd).
