# Supplementary material for: Key transcriptional effectors of the pancreatic acinar phenotype and oncogenic transformation
Source: PLoS One. 2023 Oct 5;18(10):e0291512. doi: 10.1371/journal.pone.0291512 (PMC10553828; doi:10.1371/journal.pone.0291512)
Supplement: S5 Table — (PDF) [file pone.0291512.s013.pdf]

S5 Table. Quantification of regulated genes for each dTF from ChIPseq and RNAseq results.

	ChIPseq Results			
	**Total**	**in Active Regulatory Domains**		
**dTF**	**# peaks**	**# peaks**	**% peaks**	**# genes**
Ptf1a	25,591	12,720	49.7	9,172
Nr5a2	21,284	6,936	32.6	5,743
Foxa2	19,664	5,696	29.0	5,085
Gata4	24,500	8,479	34.6	7,046

	RNAseq Results			
	**Total**	**dTF Regulated genes**		
**cKO**	**# DE genes**	**# / % w ARD**	**Down # / % w ARD**	**Up # / % w ARD**
Ptf1a	3,334	**2160 / 64.8**	1215 / 69.2	945 / 59.9
Nr5a2	1,241	**679 / 54.7**	457 / 66.5	222 / 40.1
Foxa2	2,096	**918 / 43.8**	519 / 50.5	399 / 37.4
Gata4	1,428	**776 / 54.3**	347 / 51.5	429 / 56.9

Quantification for the four dTFs of the numbers of ChIPseq peaks in normal pancreas, genes associated with the peaks by GREAT, peaks in chromatin Active Regulatory Domains (ARDs; total number of ARDs in pancreatic chromatin = 24,337), and genes with peaks in ARDs. Also tabulated are the numbers of differentially expressed (DE) genes for each of the four dTF cKOs (by RNAseq), those DE genes with dTF peak(s) in an ARD, and the breakdown for genes regulated down or up.
