# Supplementary material for: Key transcriptional effectors of the pancreatic acinar phenotype and oncogenic transformation
Source: PLoS One. 2023 Oct 5;18(10):e0291512. doi: 10.1371/journal.pone.0291512 (PMC10553828; doi:10.1371/journal.pone.0291512)
Supplement: S6 Table — (PDF) [file pone.0291512.s014.pdf]

S6 Table. Calculation of acinar deDifferentiation Indices for the dTF cKOs.

Acinar Process	# of genes	Max score*	deDifferentiation scores for the dTF-cKOs Ptf1a	Nr5a2	Foxa2	Gata4	Foxa2+Gata4
Secretory Proteins	34	100	88	44	38	18	85
Acinar Restricted	23	100	74	48	57	17	87
Differentiation	300	100	28	6	-3	0	31
Protein Synthesis & Processing	524	100	33	-1	7	11	43
Metabolism	1384	100	22	11	15	9	37
Intermediary Metabolism	56	100	79	25	16	9	74
Combined Score		**600**	**324**	**133**	**130**	**64**	**357**

* Percent of the genes in the process altered by the cKO.
