# Supplementary material for: Key transcriptional effectors of the pancreatic acinar phenotype and oncogenic transformation
Source: PLoS One. 2023 Oct 5;18(10):e0291512. doi: 10.1371/journal.pone.0291512 (PMC10553828; doi:10.1371/journal.pone.0291512)
Supplement: S7 Table — (PDF) [file pone.0291512.s015.pdf]

S7 Table. Comparison of the results of differential expression analyses for previous and new Ptf1a-cKO and Nr5a2-cKO RNAseq datasets.

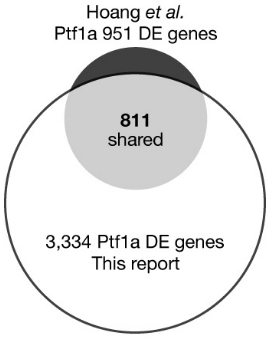

A. Ptf1a-cKO. Numbers of differentially expressed genes.

**Total # Ptf1a-cKO DE genes**		**# genes Down-regulated**		**# genes Up-regulated**	
**Previous***	**New@**	**Previous**	**New**	**Previous**	**New**
951<br>(85% shared)	3,334<br>(3.5-fold more)	517	1,757	434	1,577

* Hoang et al. *Mol Cell Biol* **36**:3033 (2016) PMID:27697859.
@ this report.

B. Ptf1a-cKO. Pathways enriched with differentially expressed genes.

		**adj p-values**					
		**New (this report)@**			**Hoang *et al.*, 2017***		
	# DE genes:	3,334	1,757	1,577	951	517	434
**Categories**	**Pathways**	**Total**	**Down**	**Up**	**Total**	**Down**	**Up**
Protein production, processing & export							
	Metabolism of proteins	2E-19	2E-10	2E-03			
	Protein processing in the ER	1E-12	1E-18		7E-08	1E-09	
	SRP-dependent import into ER	1E-09					
	Post-translational modification	4E-05		3E-03			
	N-glycan biosynthesis/trimming	5E-05	1E-07			2E-03	
	Asparagine N-linked glycosylation	2E-04	3E-05		5E-03		
	Protein export	4E-04	2E-06			8E-03	
	Membrane Trafficking	1E-03					
	Protein digestion & absorption	2E-03	2E-03		6E-07	2E-07	
	Pancreatic secretion		1E-03			4E-03	
Protein Synthesis & Translation							
	Translation	6E-14	3E-15				
	Cytoplasmic ribosomal proteins	8E-14	5E-23				
	Cytosolic tRNA aminoacylation	1E-11	1E-13				
	Cap-dependent initiation	1E-09	4E-16				
	Ribosome	4E-09	1E-15				
	Ribosome scanning & start codon recognition	9E-09	1E-11				
	Formation of the initiation complex	1E-08	1E-11				
	Translation factors	7E-06	1E-08				
	Translation elongation	4E-05			1E-04	2E-03	
	Amino acid synthesis & interconversion	1E-04	1E-03				
	Recycling of eIF2:GDP	1E-04	1E-06				
Control of Translation							
	3' UTR mediated regulation	6E-07					
	Nonsense mediated decay	3E-06					
	Insulin signaling	3E-04	7E-06	9E-03			
	mTOR signaling		5E-04				
Amino Acid Metabolism							
	Metabolism of amino acids	9E-06	3E-08		6E-09	1E-08	
	Gly, Ser & Thr metabolism	8E-05	2E-05				
	Arg & Pro metabolism	9E-05	2E-05				
RNA Metabolism						5E-05	
	Metabolism of mRNA	2E-05		7E-06			
	Metabolism of RNA	2E-05					
Metabolism		2E-19	2E-04	4E-17	2E-13		2E-10
	Lipids & lipoproteins	1E-06		1E-12	8E-05		1E-04
	Fatty acid biosynthesis	1E-05		9E-04			
	Glutathione & one carbon	7E-05	6E-04	2E-03			
	Pyrimidine	3E-04	3E-04		3E-03	2E-03	
	Urea cycle and amino groups	8E-04	2E-06		2E-03		5E-04
	Phospholipid metabolism	3E-03		9E-07			
	Peroxisome	4E-03		2E-04			4E-03
	Fatty acid oxidation	6E-03	7E-04				2E-03
Apoptosis		6E-04		1E-04	5E-08		8E-03

38 (new) versus 10 (Hoang et al. 2016) = 3.8-fold more pathways enriched

S7 Table, cont'd. Comparison of the results of differential expression analyses for previous and new Ptf1a-cKO and Nr5a2-cKO RNA-seq datasets.

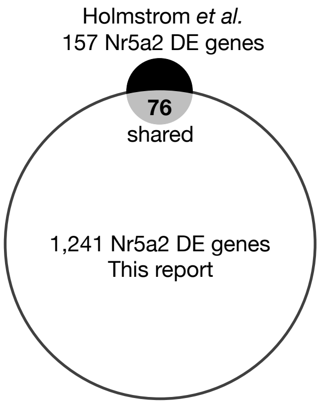

C. Nr5a2-cKO. Numbers of differentially expressed genes.

Total # Nr5a2-cKO DE genes		# genes Down-regulated		# genes Up-regulated	
**Previous[#]**	**New[&]**	**Previous**	**New**	**Previous**	**New**
157<br>(48% shared)	1,241<br>(8-fold more)	108	687	49	554

[#] Holmstrom et al. *Genes Dev* **25**:1674 (2011) PMID:21852532.
[&] this report.

D. Nr5a2-cKO. Pathways enriched with differentially expressed genes.

		adj. p values	
		**New[&]**	**Previous[#]**
**Categories**	**Pathways** Total # genes:	1,241	157
Protein production, processing & export			
	Pancreatic secretion	7E-06	2E-05
	Metabolism of proteins	3E-05	
	Protein digestion and absorption	1E-04	6E-03
	Post-translational protein modification	9E-03	
	AKT/mTOR signaling	6E-04	
Translation and control			
	Metabolism of amino acids & derivatives		4E-05
	Amino acid synthesis & interconversion	7E-04	1E-04
	Insulin signaling pathway	5E-03	
	IRS-mediated signaling	9E-03	
Metabolism		5E-11	
	Urea cycle & amino group metabolism		2E-05
	Fatty acid biosynthesis	7E-05	
	Lipids & lipoproteins	2E-04	
	Metabolism of polyamines		2E-04
	Creatine biosynthesis		4E-04
	Fatty acid, triacylglycerol, & ketone body	7E-04	
	Pyruvate	2E-03	
	Biotin transport & metabolism	4E-03	
	Sphingolipid metabolism	5E-03	
	Propanoate metabolism	5E-03	
	Retinol metabolism	5E-03	
	Branched-chain amino acid catabolism	5E-03	
	Galactose metabolism	6E-03	
Signaling			
	Signal transduction of S1P receptor	7E-05	
	Delta-Notch pathway	1E-04	
	Adipocytokine pathway	1E-03	
	EGFR1 Pathway	1E-03	
	PI3K Cascade	2E-03	

24 (new) versus 7 (Holmstrom et al.) = 3.4-fold more pathways enriched

[#] Re-analysis of the Holmstrom et al. DE gene list used ConsensusPathDB-mouse (http://cpdb.molgen.mpg.de/MCPDB)
Herwig et al. *Nat Protoc* **11**:1889 (2016) PMID:27606777.

S7 Table. The incorporation of several additional RNAseq datasets for the control, Ptf1a-cKO and Nr5a2-cKO pancreases greatly increased the numbers of differentially expressed genes detected and enriched pathways identified. 3.5-fold more genes and 3.8-fold more pathways for the Ptf1a-cKO. 8-fold more genes and 3.4-fold more pathways for the Nr5a2-cKO.
