# Supplementary material for: Key transcriptional effectors of the pancreatic acinar phenotype and oncogenic transformation
Source: PLoS One. 2023 Oct 5;18(10):e0291512. doi: 10.1371/journal.pone.0291512 (PMC10553828; doi:10.1371/journal.pone.0291512)
Supplement: S8 Table — (PDF) [file pone.0291512.s016.pdf]

S8 Table. Antibodies

PRIMARY ANTIBODIES

**Antigen**	**Host**	**Dilution**	**Source**
Immunofluorescence			
Amylase	Rabbit	1:1000	R. MacDonald et al. (1977) JBC 252: 5522.
Carboxypeptidase A	Goat	1:300	R&D Systems, AF2765
Caspase-3	Rabbit	1:100	Cell Signaling, #9661
Cela1	Rabbit	1:200	MyBioSource, MBS2093372
Cela2a	Rabbit	1:1200	abbexa, abx129880
CD45	Rat	1:400	BD Pharmingen, 553076
Claudin 18	Rabbit	1:2000	ThermoFisher, 700178
Cytokeratin 19	Rat	1:200	DSHB, TROMA III
Dclk1	Rabbit	1:200	abcam, ab37994
DsRed	Rabbit	1:1000	Clontech, 632496 (for tdTomato)
E-cadherin	Mouse	1:300	BD Transduction Laboratories, #610181
Foxa2	Rabbit	1:400	Seven Hills Bioreagents, WRAB-FOXA2
Gamt	Rabbit	1:200	Novus, NBP2-14036
Gatm	Rabbit	1:100	Novus, NBP1-89211
Gata4	Rabbit	1:100	Santa Cruz, sc-9053
Insulin	Guinea Pig	1:1000	Millipore, 4011-01
Ki67	Rabbit	1:100	abcam, ab15580
Mcm2	Rabbit	1:1000	Cell Signaling, #3619
Muc5ac	Mouse	1:40,000	Thermo Scientific, #MS-145-P0
Nr5a2	Mouse	1:250	R&D Systems, PP-H2325-00
Phospho-eIF4g	Rabbit	1:400	Cell Signaling, #2441
Phospho-MAPK	Rabbit	1:200	Cell Signaling, #4370
Phospho-Srp6	Rabbit	1:1000	Cell Signaling, #3985
Ptf1a	Rabbit	1:1000 w/TSA	BCBC Ab core, AB2153 (2432A); PMID: 18347078,<br>Hald et al., (2008) *J. Histochem. Cytochem.* **56**:587.
Rnase1	Rabbit	1:100	Bioworld, BS7958
Sox9	Rabbit	1:2000	Chemicon, AB5535
Synaptophysin 1	Guinea Pig	1:300	Synaptic Systems, #101 004
Vav1	Rabbit	1:100	Cell Signaling, #2502
Chromatin immunoprecipitation			
Ptf1a #1	Rabbit	polyclonal	Rose et al. (2001) *J. Biol. Chem.* **276**:44018; PMID: 11562365
Ptf1a #2	Rabbit	polyclonal	J. Johnson lab, Dept. Neuroscience, UT Southwestern Med Ctr
Nr5a2 #1	Guinea pig	polyclonal	Lee et al. (2008) *Mol. Endocrinol.* **22**:1345; PMID: 18323469
Nr5a2 #2	Mouse	monoclonal	R&D Systems, PP-H2325-00
Foxa2 #1	Goat	polyclonal	Santa Cruz, sc-6554x
Foxa2 #2	Rabbit	polyclonal	Seven Hills Bioreagents, WRAB-FOXA2
Gata4 #1	Rabbit	polyclonal	Santa Cruz, sc-9053x
Gata4 #2	Mouse	monoclonal	Santa Cruz, sc-25310x
H3K4me2	Rabbit	polyclonal	Millipore, #07-030
RNA polymerase II	Mouse	monoclonal	Millipore #05-623
