# Supplementary material for: Key transcriptional effectors of the pancreatic acinar phenotype and oncogenic transformation
Source: PLoS One. 2023 Oct 5;18(10):e0291512. doi: 10.1371/journal.pone.0291512 (PMC10553828; doi:10.1371/journal.pone.0291512)
Supplement: Attachment — Submitted filename: Response to Reviewers.docx [file pone.0291512.s017.docx]

Response to Reviewers

We thank the reviewers for their expertise and helpful desire to improve the manuscript.

We have responded to all the reviewers’ comments, below.

REVIEWER 1

1. *“While the identification of the four TFs is explained really well in the results, an explanation of the study design at the start of the results section will help the flow of the manuscript tremendously. This can be added as text or as a flow chart in the supplementary figures.*”

As requested by this reviewer, we add a supplemental figure (Figure S1) that outlines the decision tree to identify the four differentiation TFs and the flow/rationale for analyses of the roles they play in defining pancreatic acinar identity and differentiation status.

*2. Figures 1c and 1g are missing axis labels and need an explanation of what the change values signify is this a fold change value?*

We have added notations in the legend of Figure 1 that clarify that the axes represent the “# of genes” affected by each dTF cKO.

3. *The transition in between the various sectons of the results secton is rather abrupt and requires significant changes to help readers follow the study. As an example, the switch from study metabolic differences to transcriptomics has no explanation of transition.*

We have improved the transition between metabolic studies to transcriptional regulation by inserting two transition sentences at the end of the Metabolism section:

To summarize the transcriptomic results: the four dTFs control a very large fraction of the acinar transcriptome by activating the expression of acinar specific genes and by selectively altering the expression of many commonly expressed genes to empower specific cellular processes greatly enhanced in acinar cells. Indeed, the specialized acinar cell functions demand high metabolic activity. To delve into the nature of their collaborative regulatory strategies, we next analyzed the genetic interactions among the dTF genes and examined the role their binding plays at the transcriptional enhancer of a key target gene (*Bhlha15/Mist1*) as an illustrative example.

REVIEWER 2

*There were several abbreviations mentioned in the main text or figure legend without full*

*names (EST, MDS, and CPDB); I would advise the authors to include the full name.*

We have corrected these oversights.

In response to the Journal Requirements #2, we have removed the phrase “data not shown”, because these data are not a core part of the research in this study.
